# Large‐Scale, Abrasion‐Resistant, and Solvent‐Free Superhydrophobic Objects Fabricated by a Selective Laser Sintering 3D Printing Strategy

**DOI:** 10.1002/advs.202207183

**Published:** 2023-01-20

**Authors:** Zhenhua Wu, Congcan Shi, Aotian Chen, Yike Li, Shuang Chen, Dong Sun, Changshun Wang, Zhufeng Liu, Qi Wang, Jianyu Huang, Yamei Yue, Shanfei Zhang, Zichuan Liu, Yizhuo Xu, Jin Su, Yan Zhou, Shifeng Wen, Chunze Yan, Yusheng Shi, Xu Deng, Lei Jiang, Bin Su

**Affiliations:** ^1^ State Key Laboratory of Material Processing and Die & Mold Technology School of Materials Science and Engineering Huazhong University of Science and Technology Wuhan Hubei 430074 China; ^2^ State Key Laboratory of Advanced Electromagnetic Engineering and Technology School of Electrical and Electronic Engineering Huazhong University of Science and Technology Wuhan 430074 China; ^3^ Faculty of Engineering China University of Geosciences Wuhan Hubei 430074 China; ^4^ Institute of Fundamental and Frontier Sciences University of Electronic Science and Technology of China Chengdu 611731 China; ^5^ Key Laboratory of Bio‐inspired Materials and Interfacial Science Technical Institute of Physics and Chemistry Chinese Academy of Sciences Beijing 100190 China

**Keywords:** 3D printing, abrasion‐resistant, large‐scale, selective laser sintering, superhydrophobic

## Abstract

Manufacturing abrasion‐resistant superhydrophobic matters is challenging due to the fragile feature of the introduced micro‐/nanoscale surface roughness. Besides the long‐term durability, large scale at meter level, and 3D complex structures are of great importance for the superhydrophobic objects used across diverse industries. Here it is shown that abrasion‐resistant, half‐a‐meter scaled superhydrophobic objects can be one‐step realized by the selective laser sintering (SLS) 3D printing technology using hydrophobic‐fumed‐silica (HFS)/polymer composite grains. The HFS grains serve as the hydrophobic guests while the sintered polymeric network provides the mechanical strength, leading to low‐adhesion, intrinsic superhydrophobic objects with desired 3D structures. It is found that as‐printed structures remained anti‐wetting capabilities even after undergoing different abrasion tests, including knife cutting test, rude file grinding test, 1000 cycles of sandpaper friction test, tape test and quicksand impacting test, illustrating their abrasion‐resistant superhydrophobic stability. This strategy is applied to manufacture a shell of the unmanned aerial vehicle and an abrasion‐resistant superhydrophobic shoe, showing the industrial customization of large‐scale superhydrophobic objects. The findings thus provide insight for designing intrinsic superhydrophobic objects via the SLS 3D printing strategy that might find use in drag‐reduce, anti‐fouling, or other industrial fields in harsh operating environments.

## Graphical abstract

The abrasion‐resistant, half‐a‐meter scaled superhydrophobic objects can be one‐step realized by layer‐by‐layer accumulation of superhydrophobic thin‐films through selective laser sintering 3D printing technology. The intrinsic superhydrophobicity of the as‐prepared large‐scale objects not only makes them tolerant to mechanical abrasion, but also opens opportunities for large‐scale applications, such as the shell of aerial vehicle and the abrasion‐resistant superhydrophobic shoes.

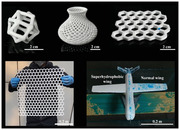

## 1 Introduction

Synthetic superhydrophobic matters have gained extensive attentions as unique interface materials with anti‐wetting, self‐cleaning and biofouling‐resistant properties in recent three decades.^[^ ^1^, ^2^, ^3^, ^4^ ^]^ Even tens of thousands of literatures focus on the fabrication of diverse superhydrophobic matters, their poor resistance to wear prevents industrial/commercial application. Generally, the introduce of micro/nanoscale roughness onto the low free energy surfaces contributes to the unique superhydrophobic capability, however, at the cost of their mechanical strength, making the surfaces fragile and highly susceptible to abrasion.

Various approaches attempt to balance the superhydrophobicity and their resistance to wear by strengthening the bonding layer^[^ ^5^, ^6^, ^7^ ^]^ or sacrificing the upper layers.^[^ ^8^ ^]^ Nevertheless, the anti‐wetting capabilities can only remain for tens of mechanical frictions. A promising strategy using the microstructural armor protecting^[^ ^9^ ^]^ can yield robust superhydrophobic surfaces. Owing to the cold/hot molding manufacturing feature of the microstructural armor, this strategy is restricted to flat surfaces, rather than complex 3D objects that are commonly used to fabricate industrial products. Furthermore, the processable size of superhydrophobic objects is of great importance. Unfortunately, the previous works^[^ ^10^, ^11^, ^12^, ^13^, ^14^ ^]^ just report the fabrication of centimeter‐scale samples, which fail to satisfy the requirement in practical fields. It is challenging to manufacture 3D complex, meter level superhydrophobic objects with the abrasion‐resistant nature.

SLS 3D printing is a mature additive manufacturing technique widely used by engineers and scientists across different industries.^[^ ^15^, ^16^, ^17^ ^]^ Its working mechanism is based on the utilization of a high‐power laser to sinter polymeric micro‐grains into 3D desired solid structure. Notably, the laser sintered polymeric network naturally provide considerable roughness. Once hydrophobic guests can be introduced into the sintered network, it is reasonably speculated that intrinsic superhydrophobicity can be constructed accordingly. Here, we report one‐step fabrication of large‐scale, abrasion‐resistant superhydrophobic objects based on the SLS 3D printing technology.

## 2 Results and Discussion

**Figure 1:**
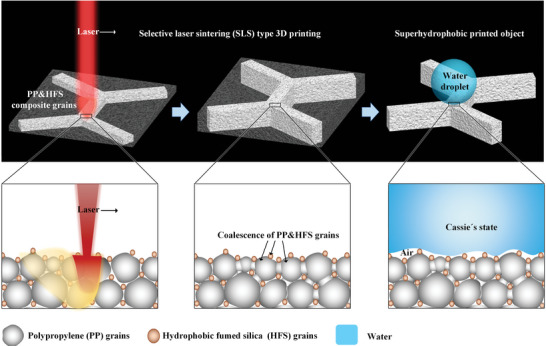
SLS type 3D printing strategy to fabricate large‐scale, abrasion‐resistant superhydrophobic architectures. A beam of high‐energy laser was selectively used on HFS/PP composite grains with a weight ratio of 4/96. PP micro‐grains would be locally fused to connect neighbors, providing considerable roughness as well as self‐supporting mechanical strength. On the other hand, nanoscale HFS grains would adhere to the molten PP grain surfaces, serving as numerous anti‐wetting points. Finally, the superhydrophobic object with desired 3D structure was generated.

**Figure** **1** conceptually shows the design mechanism of abrasion‐resistant superhydrophobicity via the SLS manufacturing technique. Primarily, 4 wt% HFS^[^ ^18^, ^19^ ^]^ grains with sizes of 20–70 nm were mixed with commercial polypropylene (PP) micro‐grains (20–100 µm, see Figure S1, Supporting Information) by a ball mill mixer. Then, the uniformly dispersed composite grains were generated, which would undergo the SLS process to generate the desired 3D structures (Figure S2, Supporting Information). During the sintering period of the laser beam, PP micro‐grains would be locally fused to connect neighbors, providing considerable roughness as well as self‐supporting mechanical strength. On the other hand, nanoscale HFS grains would adhere to the molten PP grain surfaces, serving as numerous anti‐wetting points. The SLS 3D printing is layer‐by‐layer sintering accumulation and rapid molding. Thus, another layer of composite grains with the thickness of ≈100 µm was laid on the printed architecture, repeating the similar process until the generation of the desired object.

**Figure 2:**
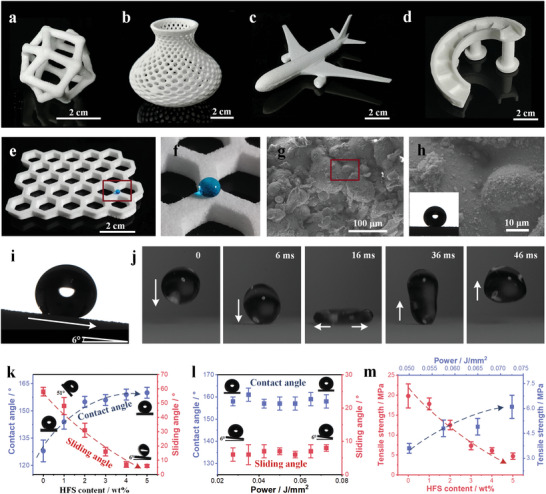
Diverse 3D printed architectures with low‐adhesion superhydrophobic performances. Examples of the printed PP/HFS superhydrophobic architectures, including a) Porous geosphere, b) Porous vase, c) Airplane, d) Spiral staircase, e) Honeycomb matrix. The printing parameters for these samples are 4 wt% HFS/PP weight ratio and a laser power density of 0.0725 J mm^−2^. f) The magnified image of (e) showing the repulsion to a dyed water droplet. g) SEM image of the surface of (f). h) The magnified image of (g), illustrating the rough micro/nano‐structures of the printed PP/HFS object (inset image, water contact angle of ≈158°). i) The water sliding angle of the surface of (f), ≈6°. j) Time‐lapse photographs of the water droplet bouncing on the surface of (f). Droplet size, ≈8 µL. k) The relation diagram of contact angle and sliding angle with the HFS content. l) The relation diagram of the contact angle and sliding angle with the laser power density. m) The relation diagram of tensile strength with the HFS content and the laser power density.

Above‐mentioned manufacturing contributes at least two advantages. The former one is that the intrinsic superhydrophobicity is abrasion‐resistant to diverse mechanical friction modes. Figure S3 (Supporting Information) shows that only one layer of the printed thin‐film was superhydrophobic and flexible. Layer‐by‐layer accumulation of such an anti‐wetting film can fabricate the inherent superhydrophobic objects, which possess porous structures (Figure S4, Supporting Information) and exhibit a good resistance to wear while maintaining the superhydrophobicity. The latter one is the rapid fabrication of the digitally designed 3D products with complex geometries (**Figure** **2**a–e) that are beyond the traditional manufacturing approaches.^[^ ^9^, ^20^, ^21^, ^22^ ^]^

Besides the diverse 3D structural architectures, the superhydrophobic capability of the printed objects was investigated. Taking the honeycomb structural sample for example (Figure 2f), a dyed water droplet remained approximately spherical on its surface, showing the static superhydrophobicity. From the scanning electron microscope (SEM) images of the sample surface (Figure 2g,h), we can find that the sintered PP rough surfaces covered by numerous HFS grains. The elemental mapping top (Figure S5, Supporting Information)/cross‐sectional (Figure S6, Supporting Information) SEM observations of the samples show that nanoscale HFS grains disperse uniformly on the sintered PP network, resulting in considerable roughness and low surface energy. This is one of the advantages of SLS 3D printing strategy. Different from traditional hot‐pressing process, SLS printed objects possess bigger roughness (Figure S7, Supporting Information). Micro‐scale PP particles and nano‐scale hydrophobic silica particles synergistically promote the excellent superhydrophobic properties. Therefore, the contact angle and the sliding angle of the sample are ≈158° (inset image of Figure 2h) and 6° (Figure 2i) respectively, illustrating the low‐adhesion superhydrophobicity. Figure 2j shows that a water droplet of ≈8 µL impacted on the printed surface with the velocity of ≈0.94 m s^−1^, and bounced instead of wetting on the superhydrophobic surface. Thus, those low‐adhesion superhydrophobic objects possess self‐cleaning property for various dirts, such as clay, grit, sawdust and concrete debris (Figure S8 and Movie S1, Supporting Information), and have good repulsion to viscous liquids including of honey, applesauce, apple tea (30 wt% applesauce), blueberry sauce, blueberry tea (30 wt% blueberry sauce) and milk (Movie S2, Supporting Information).

The dependence of the HFS content on the wettability of the printed surfaces has been studied (Figure 2k). Higher HFS content ≥5 wt% can yield a better superhydrophobicity, however, at the cost of the self‐supporting mechanical strength. On the other hand, HFS contents in the range of 2 and 3 wt% yield both high static contact angles >150° and sliding angles from ≈16° to ≈31°, indicating high‐adhesion superhydrophobicity confirmed by the bouncing tests (Figure 2k and Figure S9, Supporting Information). When the HFS content is below 1 wt%, the superhydrophobicity disappears accordingly. By tailoring the HFS content, the wettability of the printed objects is tunable (Figure S10, Movie S3 and Table S1, Supporting Information).

Laser power density is an important parameter on the performance of the printed samples in the SLS 3D printing technology. Figure 2l shows the relationship between the contact angles and sliding angles of 4 wt% HFS printed samples with diverse laser power densities. It can be observed that the laser power density mainly changes the mechanical strength, rather than wettability, of the printed objects. When the laser power density increased from 0.0500 to 0.0725 J mm^−2^, the tensile strength of the samples increased from 3.9 to 6.1 MPa (Figure 2m and Figure S11a, Supporting Information). As can be seen from cross‐sectional SEM images of samples (Figure S12, Supporting Information), the sintering joints among PP grains became larger with the increase of laser power density, resulting in greater tensile strength, which would further enhance the ability to resist external mechanical abrasion. Apart from the laser power density, the HFS content also has great influence. The tensile strength of the printed samples decreased from 19.8 to 4.7 MPa with the increase of the HFS content from 0 to 5 wt% (Figure 2m and Figure S11b, Supporting Information). In summary, the optimized parameters to print the low‐adhesion superhydrophobic objects are 4 wt% HFS content and a laser power density of 0.0725 J mm^−2^ for further study.

**Figure 3:**
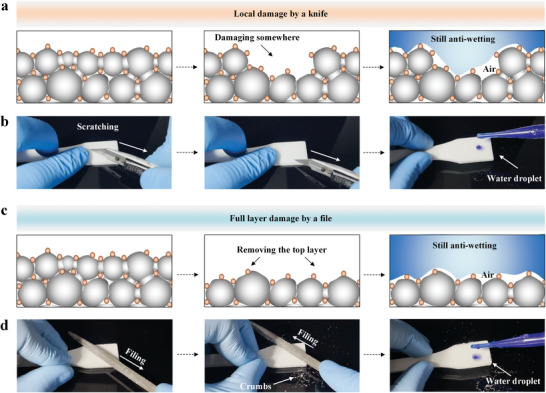
Abrasion‐resistant superhydrophobic architectures toward diverse mechanical damages. a) The superhydrophobic mechanism diagram of the printed superhydrophobic object being damaged locally. b) The optical images of the printed superhydrophobic objects being scratched by a knife and subsequent water droplet sliding off test. c) The superhydrophobic mechanism diagram of the full upper layer of the printed superhydrophobic object being damaged. d) The optical images of the printed superhydrophobic objects being filed and subsequent water droplet sliding off test. The printing parameters for these samples are 4 wt% HFS/PP weight ratio and a laser power density of 0.0725 J mm^−2^.

Although several reports demonstrate 3D printed superhydrophobic objects, they are either susceptible to abrasion^[^ ^10^, ^11^, ^14^, ^23^ ^]^ or required special chemicals,^[^ ^12^ ^]^ indicating the difficulty to survivor in harsh operating environment. Differently, our strategy is based on layer‐by‐layer accumulation of the superhydrophobic thin‐films. The sintered and connected PP skeleton of the printed superhydrophobic object plays a tenacious resistance role in the process of mechanical abrasion. Furthermore, when suffering local or lamellar violent damages (**Figure** **3**), the bared regions of the printed objects are still composed of the rough and low surface energy PP/HFS layers, indicating the abrasion‐resistant superhydrophobicity in our study.

To verify this concept, we tested the robustness of the printed superhydrophobic objects by several stability tests (Figure S13, Supporting Information), including knife cutting test (Figure 3a,b and Movie S4, Supporting Information), quicksand impacting test (Figure S14 and Movie S5, Supporting Information), jetting test (Figure S15 and Movie S6, Supporting Information), file grinding test (Figure 3c,d and Movie S7, Supporting Information), sandpaper abrasion test (Figure S16 and Movie S8, Supporting Information), tape test (Figure S17 and Movie S9, Supporting Information), fracture surface test (Figure S18 and Movie S10, Supporting Information), oily residual peeling test (Figure S19 and Movie S11, Supporting Information) and aging resistance test (Figure S20, Supporting Information).

Local damage of knife cutting was performed by deeply scratching the printed sample surface by a knife (Figure 3a,b). The damaged sites exposed anti‐wetting points, so the dyed water drops would slide off without residue, showing abrasion‐resistant superhydrophobicity (Movie S4, Supporting Information). Quicksand impacting showed the sample surface still remained the superhydrophobicity even being scoured by a sand beam with a speed of ≈4.5 m s^−1^ (Figure S14 and Movie S5, Supporting Information). For lamellar damage of rude abrasion by a file, several grinding debris can be found on the printed sample (Figure 3c,d, Supporting Information). Notably, the dyed water drops can hardly exist on the freshly exposed surfaces, showing the abrasion‐resistant ability of superhydrophobicity (Movie S7, Supporting Information). The sandpaper abrasion tests showed that, in contrast to the previous study of 40 cycles,^[^ ^21^ ^]^ water contact angles of the sample remained ≈157° even after 1000th sandpaper abrasion (Figure S16 and Movie S8, Supporting Information). From the SEM images before/after the abrasion (Figure S21, Supporting Information), it is easy to find that HFS grains remain on the fresh exposure surfaces, leading to stable superhydrophobicity. Although the molted PP surface and printed PP surface have certain roughness after sandpaper abrasion (Figure S22, Supporting Information), they are not superhydrophobic due to the lack of hydrophobic HFS particles (Figure S23, Supporting Information).

Damage of totally breaking the printed sample by hands (Figure S18a,b, Supporting Information) led to irregular fracture surface. Obviously, the dyed water drops cannot wet the damaged surfaces, indicating the intrinsic superhydrophobicity in this study (Figure S18c and Movie S10, Supporting Information). It is believed that some oily residuals might destroy the superhydrophobicity.^[^ ^21^, ^24^, ^25^ ^]^ Luckily, our samples could recover the anti‐wetting capability by peeling off the residuals and exposing fresh superhydrophobic surface instead (Figure S19 and Movie S11, Supporting Information), showing an alternative way to overcome the contamination issue. In this case, the printed superhydrophobic objects will own a long working life even being contaminated by oily residuals.

**Figure 4:**
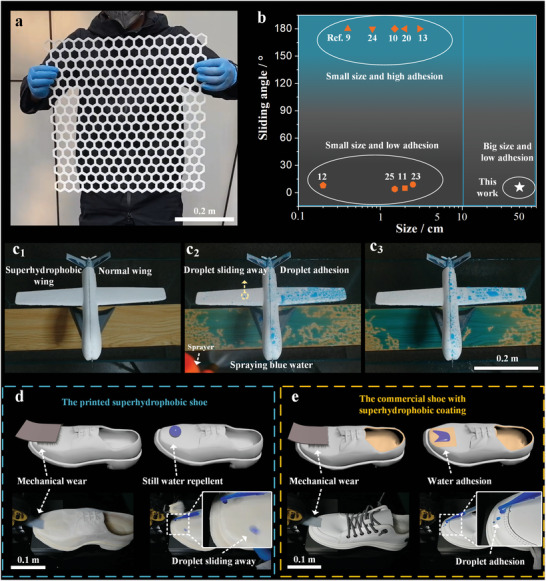
Up to half a meter scaled 3D printed superhydrophobic objects with desired structures. a) Self‐supporting honeycomb matrix with an area of 0.5 × 0.5 m^2^. b) Comparison of sliding angles and sizes of the superhydrophobic objects in this study and the reported 3D printing methods. c) Photographs of dyed rain drops on the 3D printed superhydrophobic wing shell and a normal one. The schematic diagrams and optical images of mechanical wear and anti‐wetting tests of d) a printed superhydrophobic shoe and e) a commercial shoe with a superhydrophobic coating. The fabrication details can be found in the Experimental Section.

SLS 3D printing is a mature industrial technique to fabricate objects at the meter level. Thanks to this manufacturing advance, we printed a half‐meter scaled honeycomb matrix that remained dry and self‐clean after being immersed in the blue water (**Figure** **4**a and Movie S12, Supporting Information). To the best of our knowledge, none of other literatures can fabricate abrasion‐resistant superhydrophobic objects in such a scale. Figure 4b and Table S2 (Supporting Information) compare the sizes and sliding angles of 3D printed superhydrophobic objects in this study with the existing reports.^[^ ^10^, ^11^, ^12^, ^13^, ^14^, ^23^, ^26^, ^27^, ^28^ ^]^ Previous studies use high precision of 3D printing techniques to fabricate micro/nanoscale rough surfaces, leading to high‐adhesion superhydrophobic surfaces. Even undergoing post‐treatment to reduce the surface adhesion, the sample sizes are restricted at few centimeters. Our strategy, by using the SLS 3D printing technology and composite grains, can easily increase the size of the abrasion‐resistant superhydrophobic objects to a half‐meter level. The manufacturing scale can be further enhanced by upgrading the SLS equipment.

Owing to the large‐scale, abrasion‐resistant and low‐adhesion superhydrophobic features, we demonstrate diverse 3D printed superhydrophobic architectures. We applied this strategy to manufacture a shell of the unmanned aerial vehicle (wingspan of 42 cm, fuselage of 35 cm), whose half wing was superhydrophobic and the other was normal (Figure S24 and Note S1, Supporting Information). Suffering the artificial rain drops (Figure 4c, _1_), the printed superhydrophobic wing shell remained clean, whereas several drops pinned on the other one (Figure 4c, _2__,_ _3__,_ and Movie S13, Supporting Information). Furthermore, the water‐air transmedia superhydrophobic vehicle (wingspan of 18 cm) was printed. Dyed water was difficult to adhere on the surface when the vehicle drilled out of the water (Figure S25 and Movie S14, Supporting Information). In contrast, several water stains existed on the control sample.

For practical application, we printed an abrasion‐resistant superhydrophobic shoe (Figure 4d). A home‐made cyclic abrasion setup was built (Figure S26, Supporting Information). The superhydrophobic capability of the shoe remained even after 1000th wear with the sandpaper, brush or grit. On the other hand, the commercial shoe with a superhydrophobic coating lost the superhydrophobic effect after just dozens of sanding (Figure 4e, Movie S15 and Note S2, Supporting Information).

Finally, we explored the polymer universality of this SLS printing method for superhydrophobic materials, which can be extended to polyether block amide (PEBA), polyethylene (PE), polystyrene (PS) and polymethyl methacrylate (PMMA) (Figure S27, Supporting Information). These polymers can be printed into low‐adhesion superhydrophobic materials after compounding with 4 wt% HFS grains, demonstrating the excellent universality of this printing method (Figures S28, 29 and Table S3, Supporting Information).

## 3 Conclusion

These findings demonstrate one‐step fabrication of large‐scale, abrasion‐resistant superhydrophobic objects based on the SLS 3D printing technology. Layer‐by‐layer accumulation of intrinsic superhydrophobic thin‐films enables long‐term abrasion‐resistant ability toward diverse mechanical wears, such as knife cutting, file grinding, sandpaper abrasion, tape adhesion and quicksand impacting. We used this strategy to manufacture an anti‐wetting shell of the unmanned aerial vehicle and an abrasion‐resistant superhydrophobic shoe, showing commercial potentials. Beyond PP grains, the adaption of our strategy can be extended to PEBA, PE, PS and PMMA. We believe that this study can provide a solution for the industrial application of superhydrophobic materials with desired 3D structures, and broaden their practical application range through its large‐scale, customizable design capabilities.

## Conflict of Interest

The authors declare no conflict of interest.

## Supporting information

Supporting Information

Supplemental Movie 1

Supplemental Movie 2

Supplemental Movie 3

Supplemental Movie 4

Supplemental Movie 5

Supplemental Movie 6

Supplemental Movie 7

Supplemental Movie 8

Supplemental Movie 9

Supplemental Movie 10

Supplemental Movie 11

Supplemental Movie 12

Supplemental Movie 13

Supplemental Movie 14

Supplemental Movie 15

## Data Availability

The data that support the findings of this study are available from the corresponding author upon reasonable request.
