# Supplementary material for: Large‐Scale, Abrasion‐Resistant, and Solvent‐Free Superhydrophobic Objects Fabricated by a Selective Laser Sintering 3D Printing Strategy
Source: Adv Sci (Weinh). 2023 Jan 20;10(9):2207183. doi: 10.1002/advs.202207183 (PMC10037971; doi:10.1002/advs.202207183)
Supplement: Supplementary file 1 — Supporting Information [file ADVS-10-2207183-s015.pdf]

ADVANCED SCIENCE
Open Access

Supporting Information

for *Adv. Sci.*, DOI 10.1002/advs.202207183

Large-Scale, Abrasion-Resistant, and Solvent-Free Superhydrophobic Objects Fabricated by a Selective Laser Sintering 3D Printing Strategy

*Zhenhua Wu, Congcan Shi, Aotian Chen, Yike Li, Shuang Chen, Dong Sun, Changshun Wang, Zhufeng Liu, Qi Wang, Jianyu Huang, Yamei Yue, Shanfei Zhang, Zichuan Liu, Yizhuo Xu, Jin Su, Yan Zhou, Shifeng Wen, Chunze Yan, Yusheng Shi, Xu Deng, Lei Jiang and Bin Su**

Supporting Information

Large-scale, abrasion-resistant and solvent-free superhydrophobic objects fabricated by a selective laser sintering 3D printing strategy

*Zhenhua Wu, Congcan Shi, Aotian Chen, Yike Li, Shuang Chen, Dong Sun, Changshun Wang, Zhufeng Liu, Qi Wang, Jianyu Huang, Yamei Yue, Shanfei Zhang, Zichuan Liu, Yizhuo Xu, Jin Su, Yan Zhou, Shifeng Wen, Chunze Yan, Yusheng Shi, Xu Deng, Lei Jiang, Bin Su**

Z. Wu, C. Shi, A. Chen, Y. Li, S. Chen, D. Sun, C. Wang, Z. Liu, J. Huang, Y. Yue, S. Zhang, Z. Liu, Y. Xu, J. Su, S. Wen, C. Yan, Y. Shi, B. Su
State Key Laboratory of Material Processing and Die & Mold Technology, School of Materials Science and Engineering, Huazhong University of Science and Technology, Wuhan 430074, Hubei, P. R. China
E-mail: subin@hust.edu.cn

Q. Wang
State Key Laboratory of Advanced Electromagnetic Engineering and Technology, School of Electrical and Electronic Engineering, Huazhong University of Science and Technology, Wuhan 430074, P. R. China
Y. Zhou
Faculty of Engineering, China University of Geosciences, Wuhan, Hubei 430074, China
X. Deng
Institute of Fundamental and Frontier Sciences, University of Electronic Science and Technology of China, Chengdu, China
L. Jiang
Key Laboratory of Bio-inspired Materials and Interfacial Science, Technical Institute of Physics and Chemistry, Chinese Academy of Sciences, 100190 Beijing, China

Keywords: superhydrophobic, abrasion-resistant, selective laser sintering, 3D printing, large-scale

Content

Experimental Section

Figure S1 Characterization of the PP and HFS grains and their size distribution.

Figure S2 SLS 3D printer.

Figure S3 Superhydrophobicity & flexibility of one layer of the printed PP/HFS.

Figure S4 The reconstructed 3D copy images of the 3D printed superhydrophobic objects by an in situ high-resolution X-ray Micro-CT technique.

Figure S5 Top-view SEM images and their element analysis of the surfaces of the printed objects with different HFS contents.

Figure S6 Cross-sectional SEM images and their element analysis of the printed objects with different HFS contents.

Figure S7 The 3D topographies of pure PP film, PP film + HFS coatings, the printed pure PP object and the printed PP/HFS object, illustrating their different surface roughnesses.

Figure S8 Self-cleaning of diverse dirts on the printed superhydrophobic surfaces.

Figure S9 Time-lapse photographs of water droplets bouncing on the printed objects with different HFS contents.

Figure S10 Wettability of the printed objects with different HFS contents.

Figure S11 Tensile strength-elongation curves of the printed samples.

Figure S12 Cross-sectional SEM images of the printed objects with different laser power densities.

Figure S13 Schematic diagram of different approaches for testing the abrasion-resistant superhydrophobicity of the printed samples.

Figure S14 Quicksand impacting test.

Figure S15 Jetting test.

Figure S16 Sandpaper abrasion test.

Figure S17 Tape test.

Figure S18 Fracture surface test.

Figure S19 Oily residual test.

Figure S20 The relationship between the contact angle of the printed samples and their aging time.

Figure S21 SEM images and their element analysis of the printed sample surfaces after diverse cycles of sandpaper test.

Figure S22 Comparison of 3D topographies of the melted PP surface, the printed PP surface and the printed PP/HFS surface before and after 1000th sandpaper abrasion.

Figure S23 SEM images and element analysis of the melted PP surface and the printed PP surface before and after 1000th sandpaper abrasion.

Figure S24 Schematic diagram of the airplane model assembled with the printed superhydrophobic and the normal wing shell.

Figure S25 Time-lapse photographs of the printed superhydrophobic/normal transmedia vehicles flying out of the water.

Figure S26 The home-made shoe polishing device.

Figure S27 Characterization of the PEBA, PE, PS and PMMA grains and their size distribution.

Figure S28 Top-view SEM images of the printed samples with different polymers.

Figure S29 Polymer universality of this printing method of the abrasion-resistant superhydrophobic objects, including PEBA, PE, PS and PMMA.

Note. S1 Details of the 3D printed superhydrophobic wing shell and transmedia vehicles.

Note. S2 Details of the printed superhydrophobic shoe and the commercial shoe with superhydrophobic coating.

Table S1 The dependence of wettability of the printed objects on the laser power density and the HFS content.

Table S2 Comparison of sliding angles and sizes of the printed superhydrophobic objects in this study and existing reports.

Table S3 The SLS processing parameters for polymer universality, including PEBA, PE, PS and PMMA.

Movie S1 Self-cleaning ability of the printed superhydrophobic surfaces.

Movie S2 Viscous liquid tests.

Movie S3 Wettability of the printed samples with different HFS contents.

Movie S4 Knife scratching test.

Movie S5 Quicksand impacting test.

Movie S6 Jetting test.

Movie S7 File grinding test.

Movie S8 Sandpaper abrasion test.

Movie S9 Tape test.

Movie S10 Fracture surface test.

Movie S11 Oily residual test.

Movie S12 Half meter scaled superhydrophobic printed object.

Movie S13 Anti-wetting property of the printed plane shell.

Movie S14 The printed superhydrophobic/normal transmedia vehicles flying out of the water.

Movie S15 Mechanical wear and water droplet test of the printed superhydrophobic shoe and the commercial shoe with superhydrophobic coating.

Experimental Section

Preparation of the composite grains: Hydrophobic-fumed-silica (HFS) and compounding polypropylene (PP) particles were purchased from Evonik Industries AG and Wanhua Chemical Group Co., Ltd., respectively. Polyether block amide (PEBA) particles, polyethylene (PE) particles, polystyrene (PS) particles and polymethyl methacrylate (PMMA) particles were bought from Evonik Specialty Chemicals (Shanghai) Co., Ltd, Dongguan Suzhiyuan Plastic Group Co., Ltd., Guangdong Silver Age Sci. & Tech. Co., Ltd. and Evonik Industries AG, respectively. The as-received PP (D50 = 77 μm) has a bulk density of 0.32 g/cm^3, a melting point of 149 °C and a molecular weight of ~80000, which can meet the powder spreading effect and the design of printing layer thickness. The PP/HFS composite grains with different HFS weight ratio of 0, 1, 2, 3, 4 and 5 wt% were obtained by homogeneously mixing the PP and HFS grains in a ball mill mixer with a rate of 500 rpm for 10 minutes. Other composite grains (including 4 wt% HFS) for polymer universality were prepared in the same way. Before the selective laser sintering (SLS) 3D printing, all the composite grains were sifted through 80 mesh to remove bulk flocs.

SLS 3D printing to fabricate superhydrophobic objects: All the three-dimensional models were designed using 3D max software. The 3D printing machine (HUAKE 3D S320) with a 50 W CO_2 laser was employed to print the digital models. The PP/HFS composite grains with 4 wt% HFS was chosen as the printing materials for printing a variety of complex models due to its balanced superhydrophobicity and mechanical strength. The optimal parameters for 3D printing process were as follows: the preheating temperature of 140 °C, laser power of 29 W, laser scan velocity of 4000 mm/s and the layer thickness of 0.1 mm. In terms of other PP/HFS composite grains with different HFS weight ratio, the printing parameters except the laser power remained. The specific laser energy density can be found in Table S1 below. The SLS processing parameters for polymer universality, including PEBA, PE, PS and PMMA, were

shown in Table S3. The mass fraction of all polymers was 96 wt% and the mass fraction of HFS was 4 wt%.

Characterization: A digital camera (Sony a6300) or a smartphone (POT-AL00a, Huawei) was used to record the optical images and videos. The surface and cross-sectional morphologies were observed by the field emission scanning electron microscope (FE-SEM, JSM-7600F, JEOL). The element distributions on the sample surfaces and sections were analyzed by the energy disperse spectroscopy (EDS, Oxford). The mechanical property of the sample was tested by the electronic dynamic static fatigue testing machine (E1000, ITW Instron). Transient photographs (1000 pictures per second) of the water droplet rebounding on the sample were recorded on the MEMRECAM HX-7s high-speed camera system (ST-857, NAC Image Technology Inc.) controlled by MEMRECAM HXLINK (SP-642). The water contact angle and sliding angle on the samples were measured using the contact angle meter (SDC-350, SINDIN, China). The water droplets were 5 μL for water contact angle tests and 10 μL for sliding angle tests.

Self-cleaning testing: Self-cleaning of dirts on the printed superhydrophobic surfaces were tested as follows. Clay, grit, sawdust or concrete debris were placed onto the printed superhydrophobic surfaces, respectively. Then, several dyed water droplets were dropped on the printed superhydrophobic surfaces to test the self-cleaning property (Figure S8).

Viscous liquid testing: The experiments for viscous liquids including of honey (Shanghai Guanshengyuan Bee Products Co.,Ltd), applesauce, apple tea (30 wt% applesauce), blueberry sauce (Hangzhou Kewpie Corporation), blueberry tea (30 wt% blueberry sauce) and milk (Inner Mongolia Yili Industrial Group) on the superhydrophobic surfaces were performed. Specifically, the superhydrophobic sample was first held at an angle; then the viscous liquids were then dropped onto the superhydrophobic surface. The viscous liquids slipped off and there was no residue on the superhydrophobic surface, indicating that the superhydrophobic surface has a good repulsion to the viscous liquids.

Detailed abrasion resistant tests for the superhydrophobic stability: In order to demonstrate the abrasion-resistant superhydrophobic stability of the printed objects, we carried out a series of stability tests including of knife cutting (Figure 3a,b and Movie S4), quicksand impacting (Figure S14 and Movie S5), jetting (Figure S15 and Movie S6), file abrasion (Figure 3c,d and Movie S7), sandpaper abrasion (Figure S16 and Movie S8), tape adhesion (Figure S17 and Movie S9), and fracture surface (Figure S18 and Movie S10).

1. Figure 3b shows the knife cutting test. A cutter knife (Deli 2043) was used to scratch the printed sample surface, and then dyed water droplets were dropped to verify its superhydrophobicity.
2. Quicksand impacting was performed in reference to ***Standard Test Methods for Abrasion Resistance of Organic Coatings by Falling Abrasive*** (D968–93). The schematic diagram and optical photos of the test device are shown in Figure S13b and Figure S14. 1 L fine sand (diameters ≤0.6 mm) fell from a height of 104 centimeters through a vertical hollow tube, impacting onto the printed sample surface at an angle of 45°. The speed of the fine sand impacting the sample surface was ~4.5 m/s estimated by the gravimetric calculation. After quicksand impacting, several dyed water droplets were applied to the impacted surface to verify its superhydrophobicity.
3. Figure S15 shows images of the continuous impact of a water flow on the printed sample surface. The surface of the sample remained clean after being impinged by a high-speed water flow extruded with a syringe.
4. Grinding the printed sample with a file to show its mechanical abrasion-resistant ability and superhydrophobicity (Figure 3d). First, hold the sample on the table with one hand. Then, press the file hard on the sample with the other hand and grind it back and forth for tens of cycles. Finally, drop dyed water droplets on the damaged surfaces to demonstrate its superhydrophobic stability.
5. Figure S16 shows that the abrasion-resistant ability of the printed sample was tested by a

sandpaper abrasion method. The printed sample was horizontally put onto the flocking sandpaper with different roughnesses, respectively (Flocking sandpaper, Grit No. 60, 240 and 1000 Gold Cattle). The sample was abraded 12 cm by the sandpaper under a weight of 200 g for 1000 times (Figure S16a,b). Water contact angles were measured after the 50th, 100th, 200th, 400th, 600th, 800th and 1000th abrasion tests, respectively (Figure S16c).

6. The tape test was performed in reference to ***Standard Test Methods for Measuring Adhesion by Tape Test*** (D3359-09). First, place the printed flat sample horizontally on the table. Then, stick the tape (PVC electrical insulation tape, Gongniu Group Co., Ltd.) on the sample surface and roll a weight of 500 g back and forth to make the tape fully bond with the sample (Figure S17a,b). Further, remove the tape by grabbing the free end of the tape and quickly pull it off (not jerked) at an angle of as close to 180° as possible (Figure S17c). Finally, a few dyed water droplets were added to the taped sample to verify the superhydrophobic effect (Figure S17d).
7. The damaged fracture surfaces by hands (Figure S18) were performed to demonstrate the intrinsic superhydrophobicity of the printed sample. The printed sample with 4 mm thickness was broken off forcibly, and the dyed water droplets were dropped on the fracture surfaces. The rapid traceless sliding of water droplets indicated the superhydrophobicity of the fracture surfaces.

Oily residual test: Oily residual was performed by dropping the molten paraffin with ceresin (the melting point of 52-54 °C, Aladdin industrial corporation) on the printed sample surface, and cooling naturally for a few minutes to cure (Figure S19a). A few dyed water droplets were dropped to test the superhydrophobicity after removing the oily residual (Figure S19b,c).

Aging resistance test: Aging resistance test was performed by a sunlight weather-conditions meter (FY3600$^+$, Wenzhou Fangyuan instrument Co., Ltd), and the insolation test environment was temperature of 35°C, humidity of 40%, irradiance of 42 W/m^2, irradiation range of 300-400 nm.

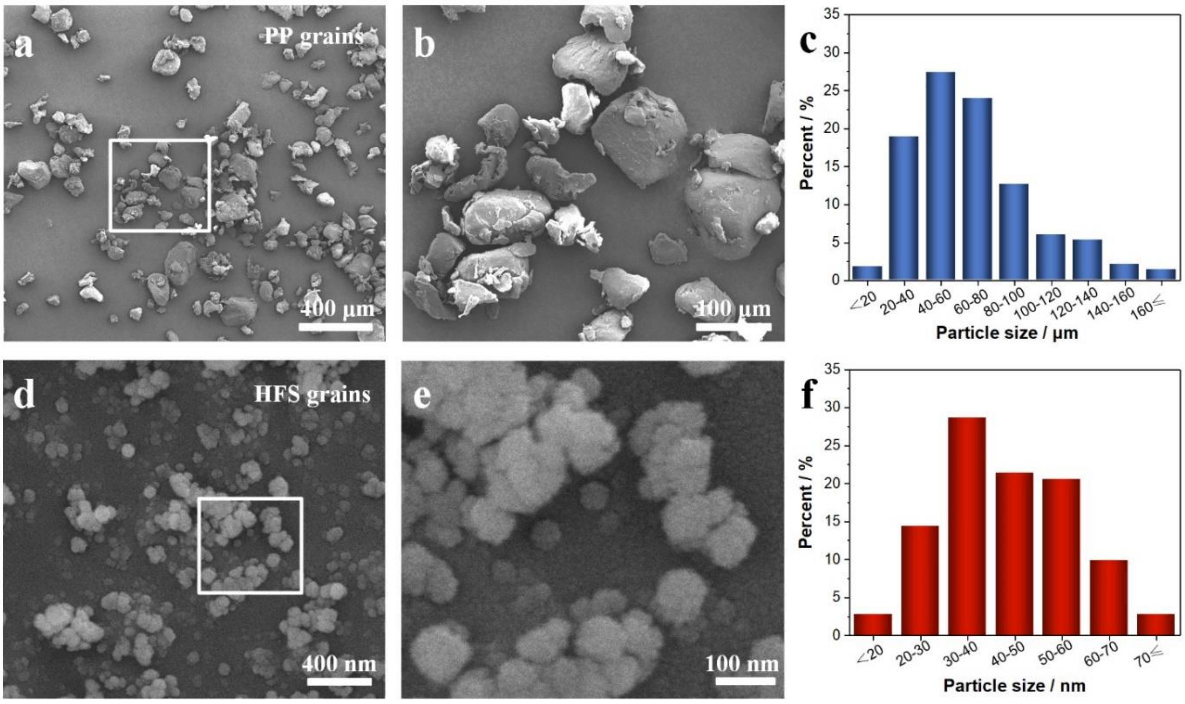

Figure S1. Characterization of the PP and HFS grains and their size distribution. a-b) SEM images of PP grains. c) Statistics of PP particle size. d-e) SEM images of HFS grains. f) Statistics of HFS particle size.

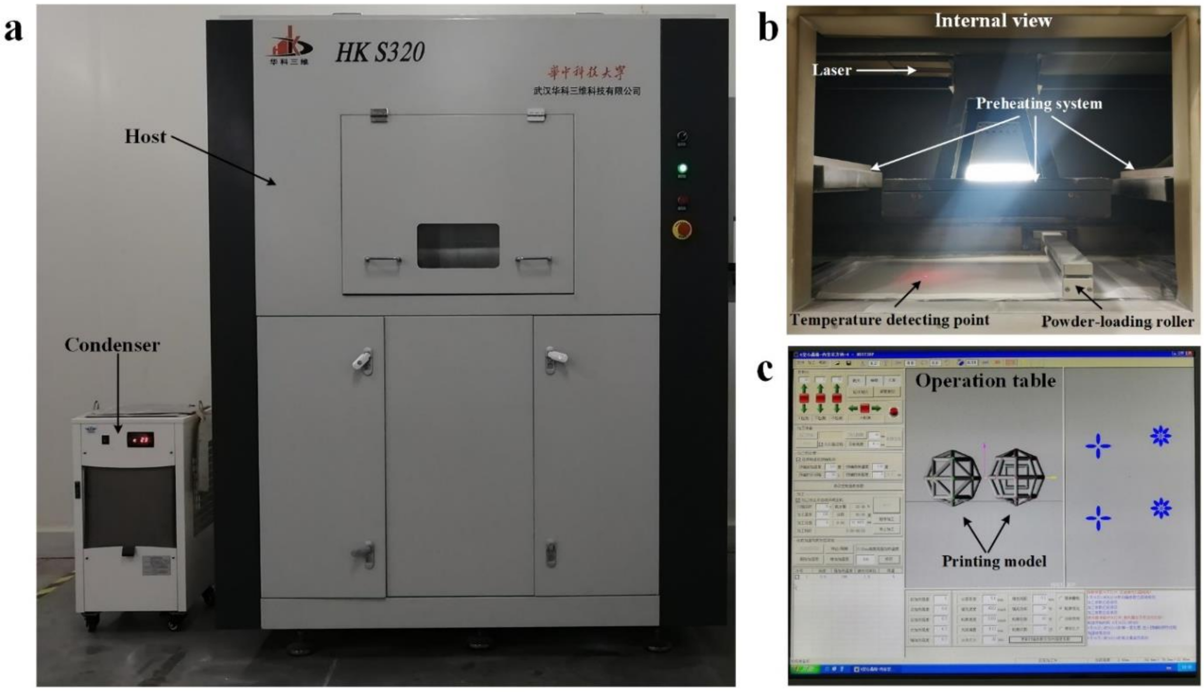

Figure S2. SLS 3D printer. Optical photos of a) the SLS 3D printing machine, b) internal working space and c) the software operation table.

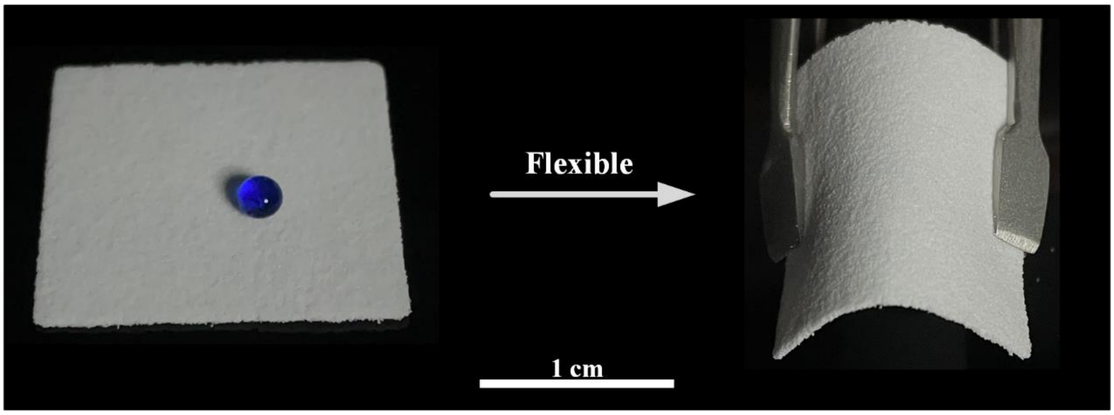

Figure S3. Superhydrophobicity & flexibility of one layer of the printed PP/HFS. Optical photos of one layer of the printed PP/HFS and its flexible state bent by the tweezers. The printing parameters are 4 wt% HFS/PP weight ratio and a laser power density of 0.0725 J/mm^2.

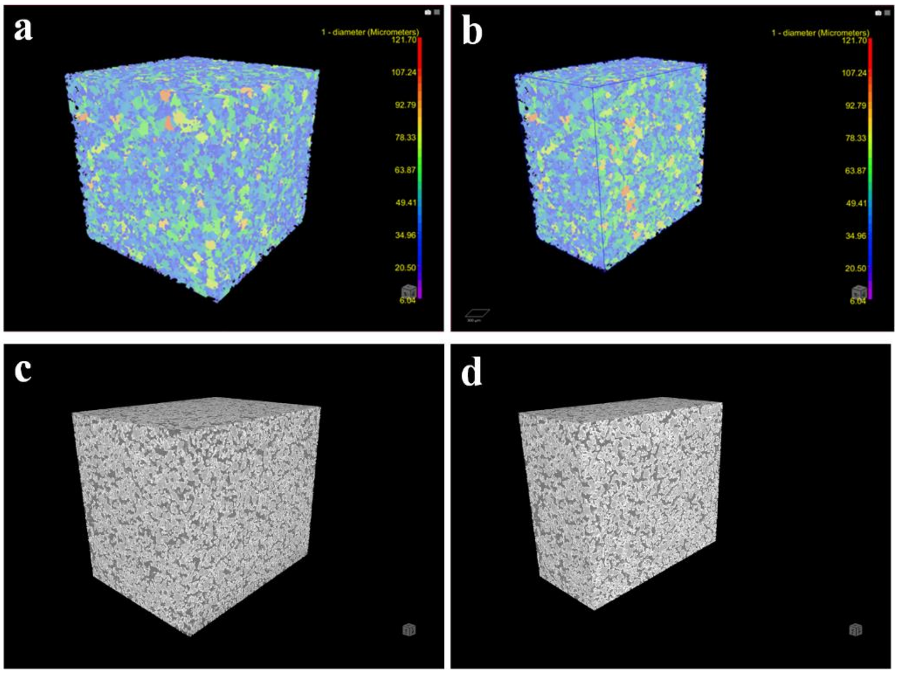

Figure S4. The reconstructed 3D copy images of the 3D printed superhydrophobic objects by an in situ high-resolution X-ray Micro-CT technique.

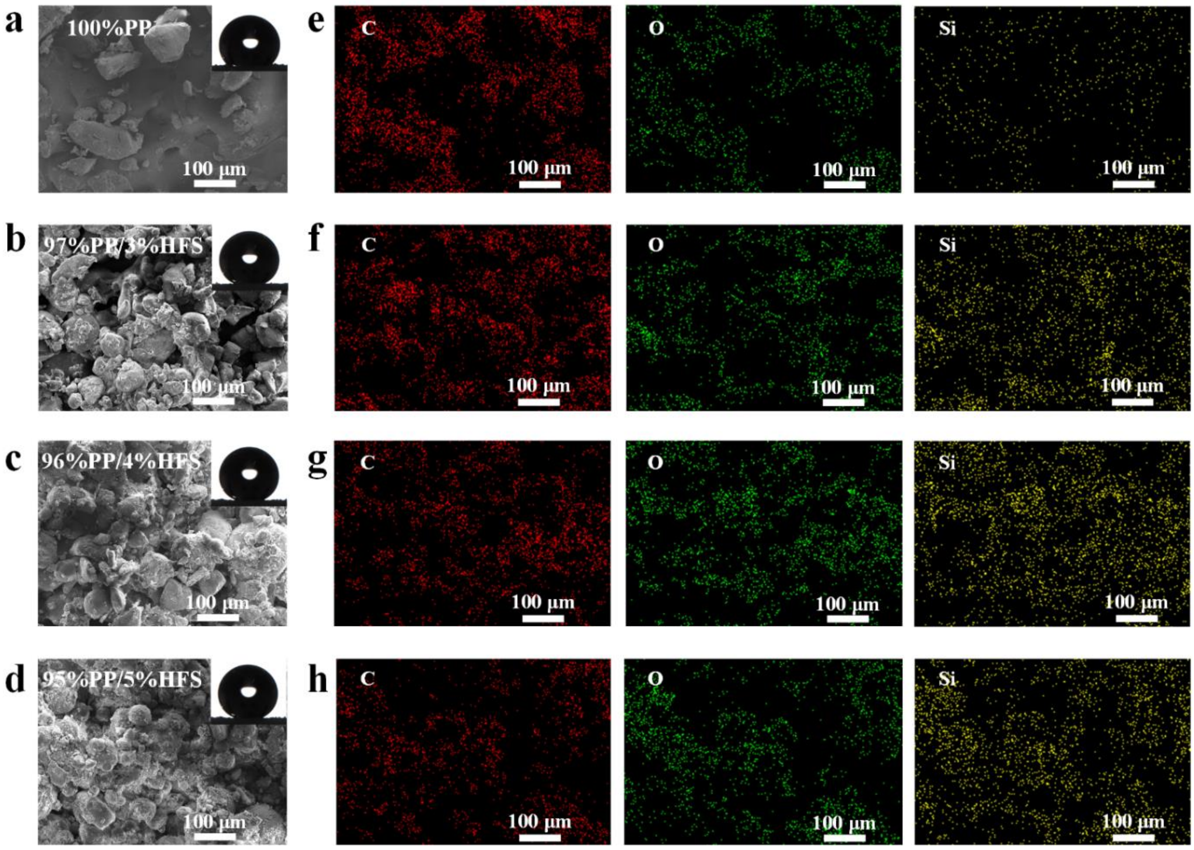

Figure S5. Top-view SEM images and their element analysis of the surfaces of the printed objects with different HFS contents. The laser power density of PP is 0.0350 J/mm^2, while the laser power densities of the composites containing 3~5 wt% HFS are 0.0725 J/mm^2.

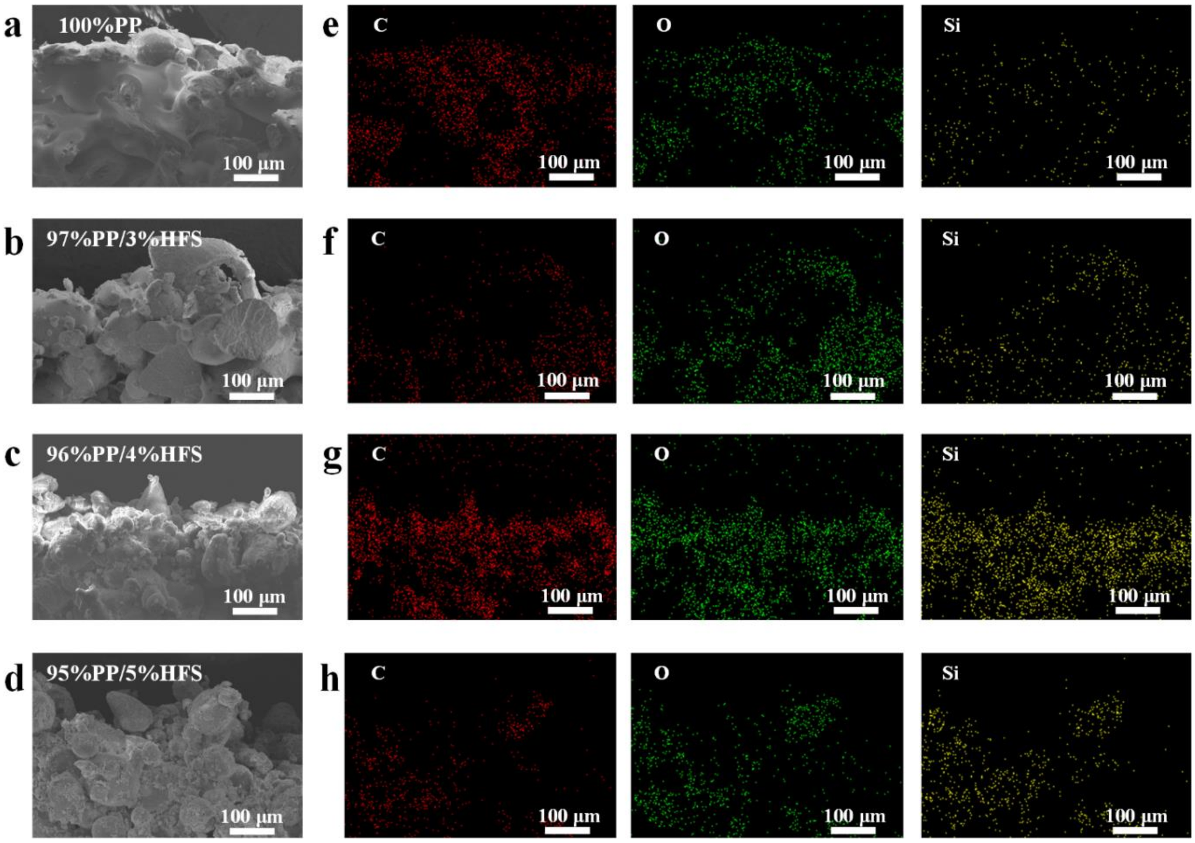

Figure S6. Cross-sectional SEM images and their element analysis of the printed objects with different HFS contents. The laser power density of PP is 0.0350 J/mm^2, while the laser power densities of the composites containing 3~5 wt% HFS are 0.0725 J/mm^2.

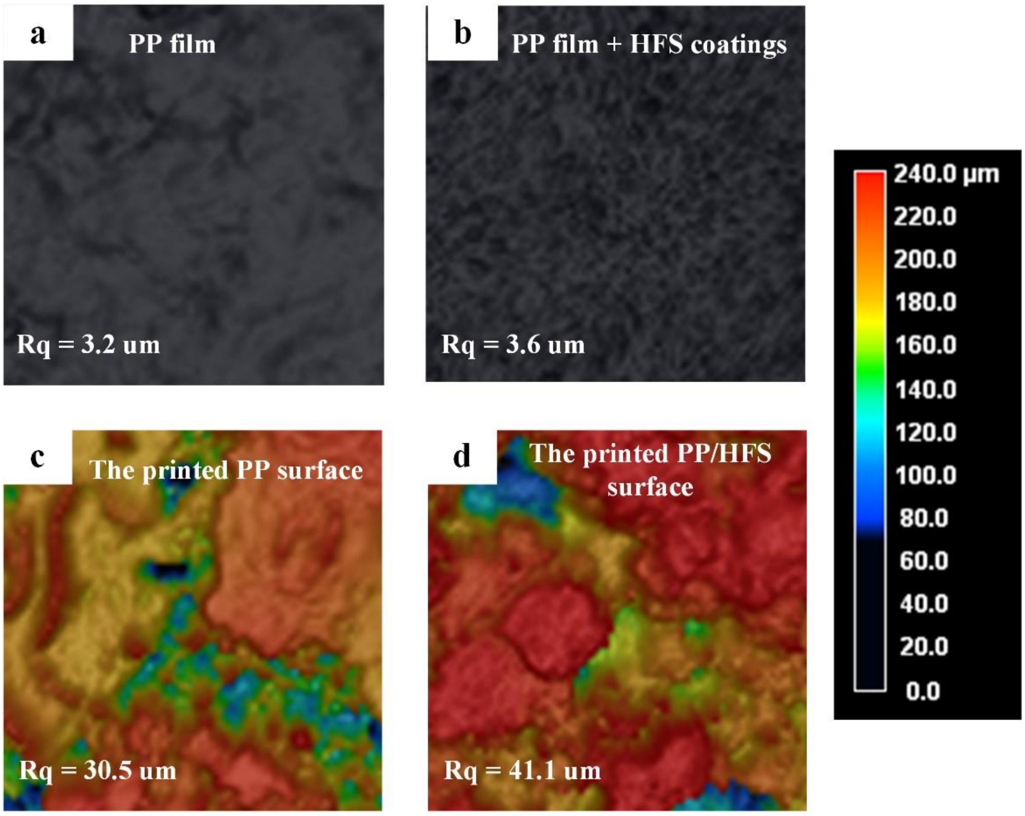

Figure S7. The 3D topographies of pure PP film, PP film + HFS coatings, the printed pure PP object and the printed PP/HFS object, illustrating their different surface roughnesses.

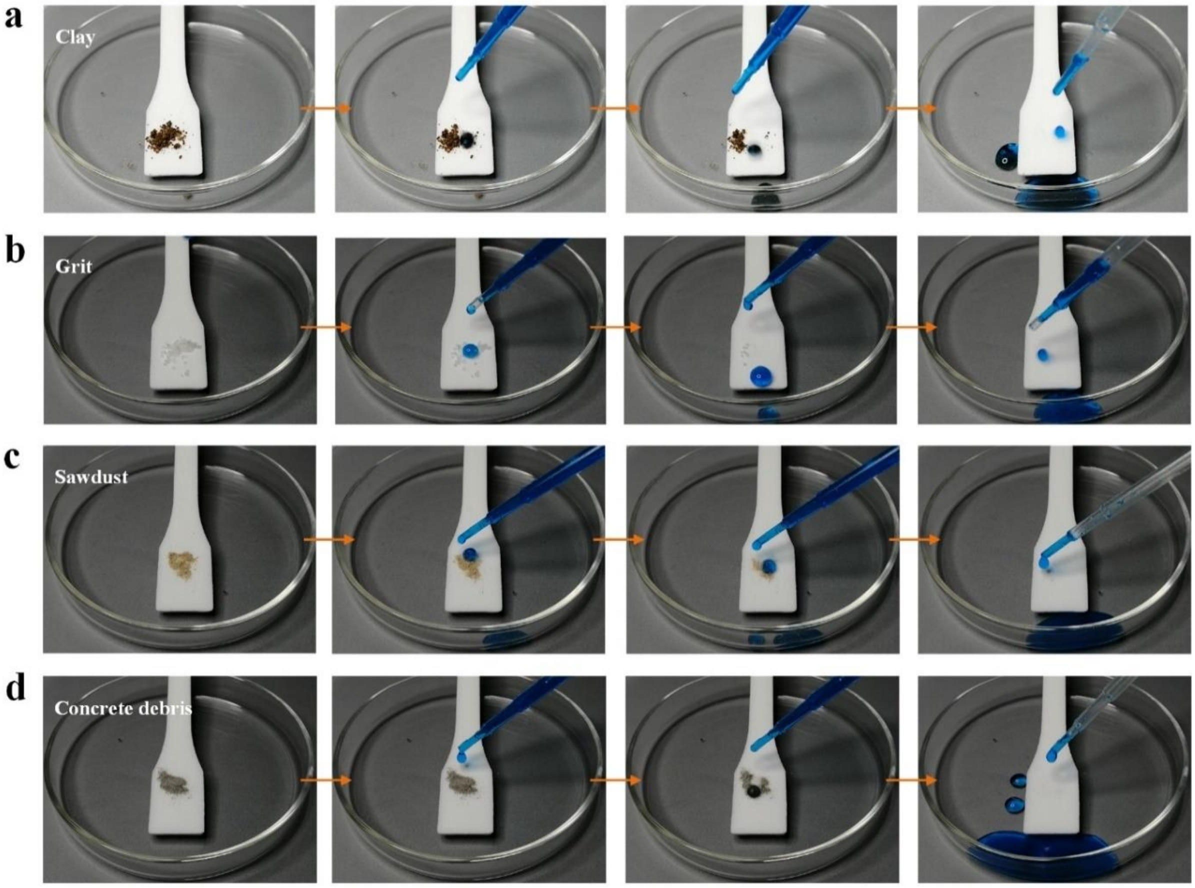

Figure S8. Self-cleaning of diverse dirts on the printed superhydrophobic surfaces. a) Clay, b) grit, c) sawdust and d) concrete debris were put onto the printed superhydrophobic surfaces. Several dyed water droplets were then spread onto the printed superhydrophobic surfaces to test the self-cleaning property. The printing parameters for these samples are 4 wt% HFS/PP weight ratio and a laser power density of 0.0725 J/mm^2.

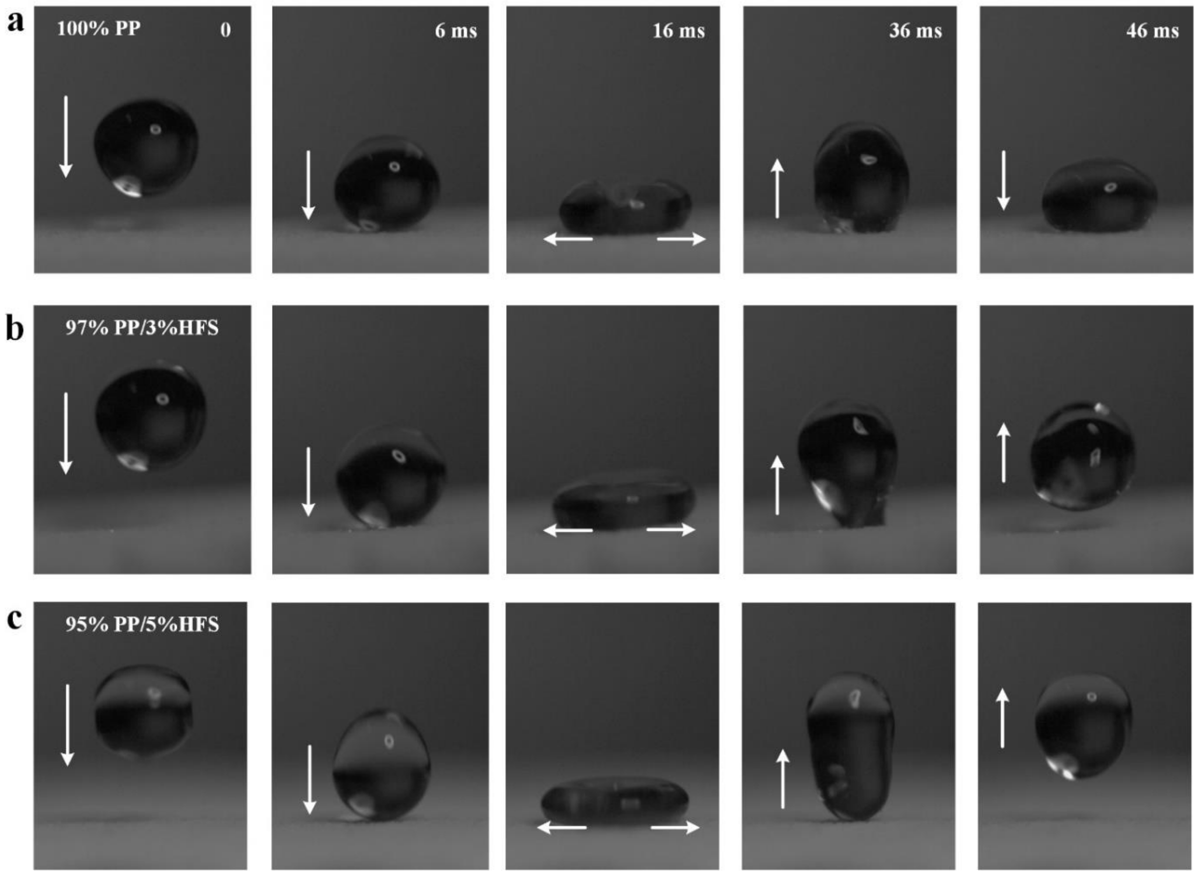

Figure S9. Time-lapse photographs of water droplets bouncing on the printed objects with different HFS contents. The water droplet volume is ~8 μL. The laser power density of PP is 0.0350 J/mm^2, while the laser power densities of the composites containing 3 wt% and 5 wt% HFS are 0.0725 J/mm^2.

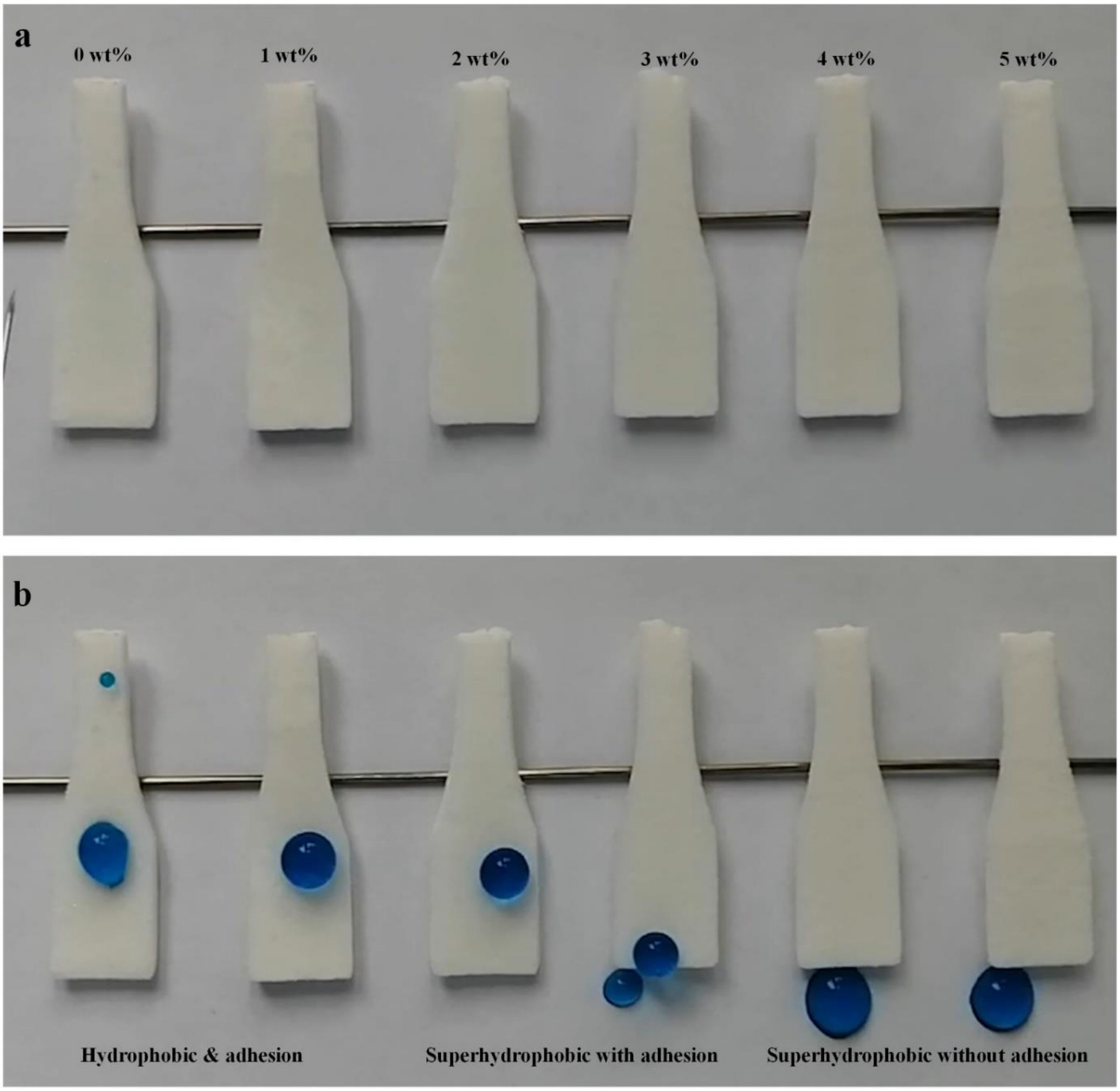

Figure S10. Wettability of the printed objects with different HFS contents. When the HFS content is below 1 wt%, the printed samples only exhibit hydrophobicity with high adhesion. Increasing the HFS content to 2-3 wt%, the printed samples become superhydrophobic, but highly adhesive. When the HFS content is increased above 4 wt%, the printed samples exhibit superhydrophobicity with low adhesion. The laser power densities of the composites containing 0~5 wt% HFS are 0.0350, 0.0425, 0.0500, 0.0725, 0.0725, 0.0725 J/mm^2, respectively.

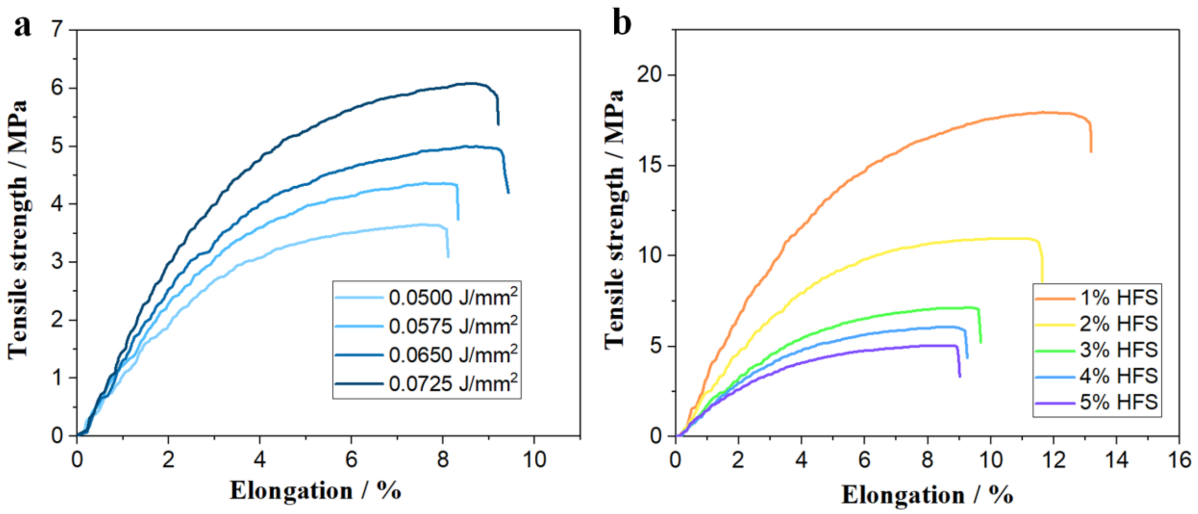

Figure S11. Tensile strength-elongation curves of the printed samples. a) Tensile strength-elongation curves with different laser power densities. The HFS contents of the samples are 4 wt%. b) Tensile strength-elongation curves with different HFS contents. The laser power densities of the composites containing 1~5 wt% HFS are 0.0425, 0.0500, 0.0725, 0.0725, 0.0725 J/mm^2, respectively.

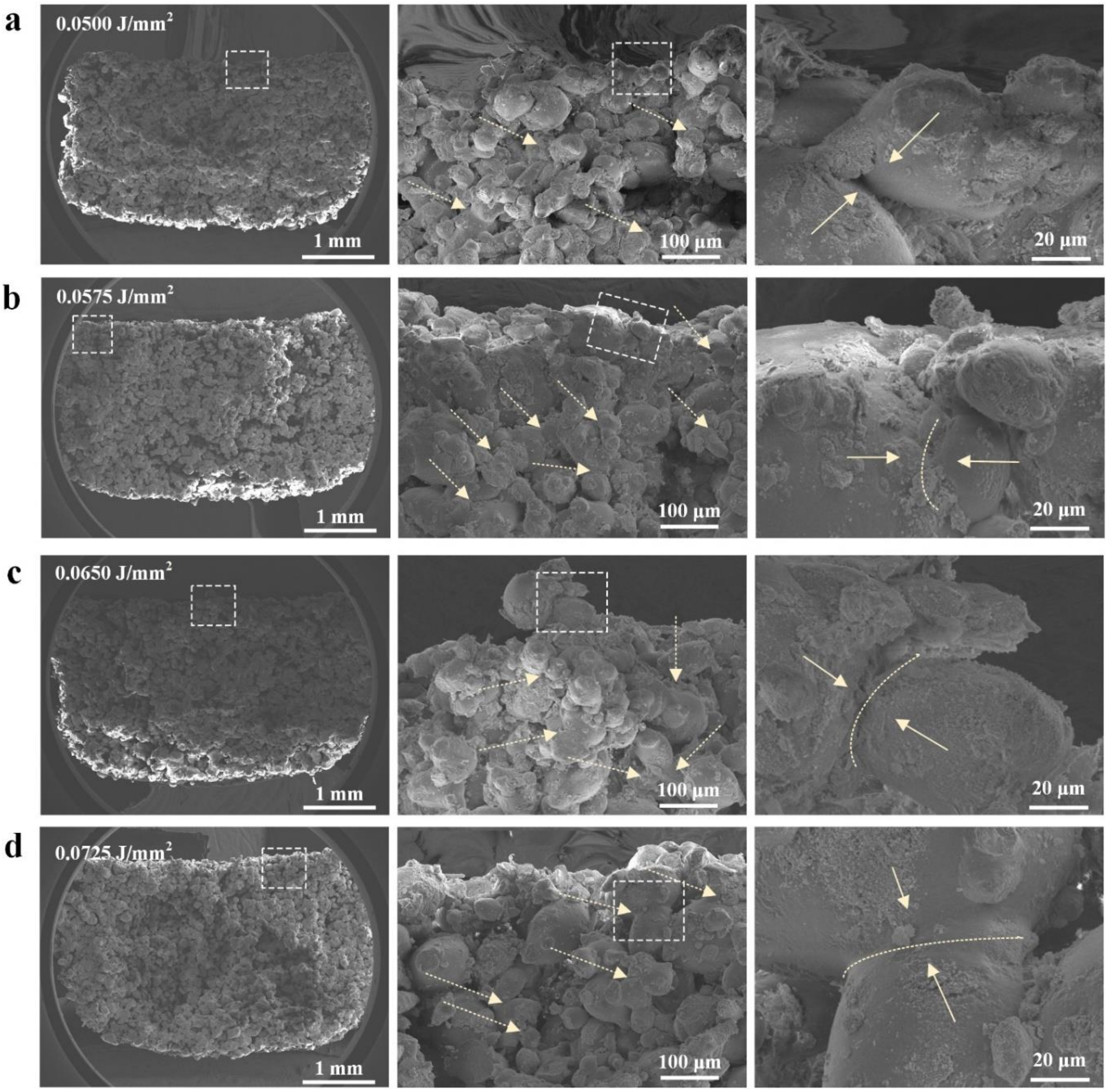

Figure S12. Cross-sectional SEM images of the printed objects with different laser power densities. The HFS contents of the samples are 4 wt%.

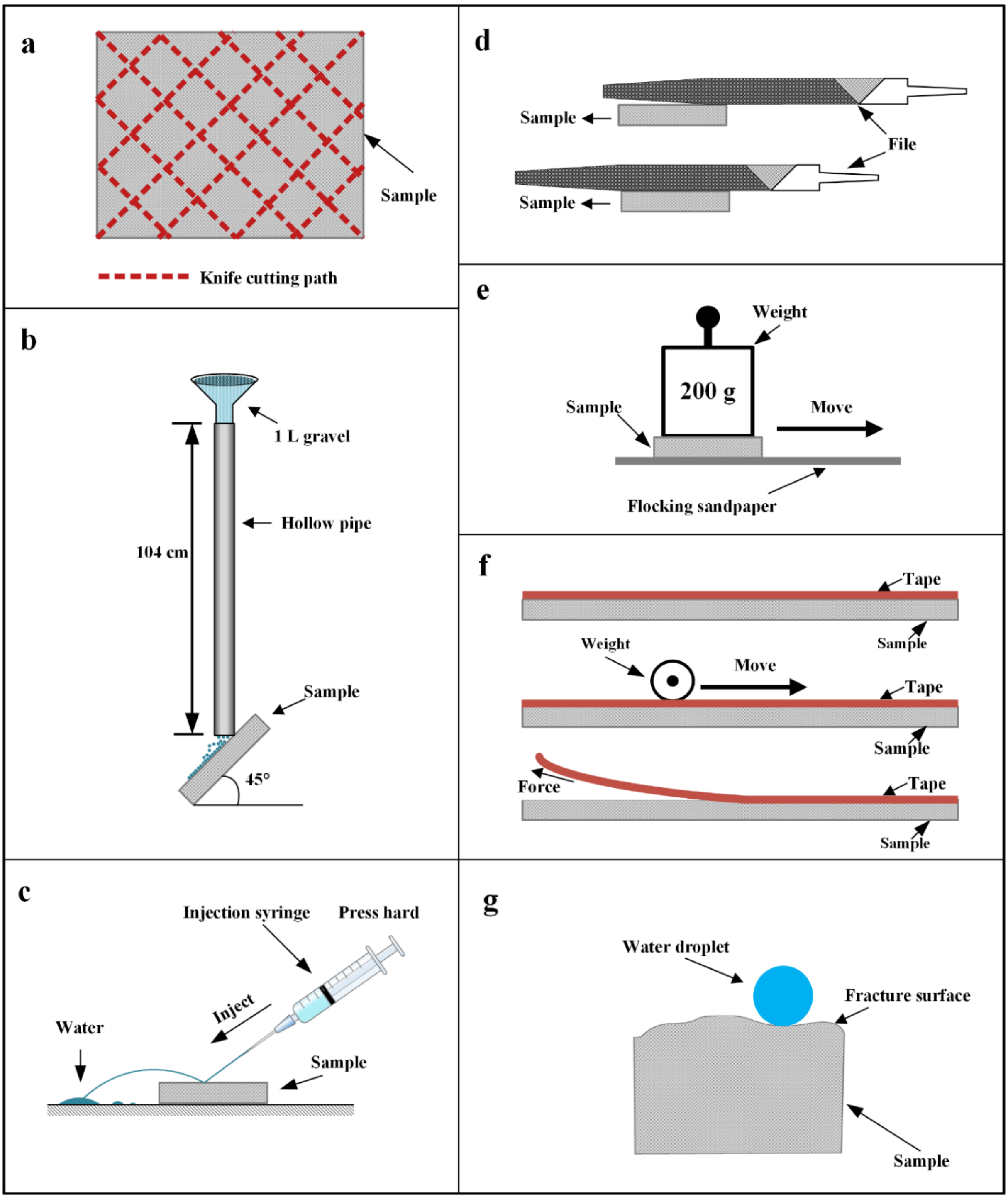

Figure S13. Schematic diagram of different approaches for testing the abrasion-resistant superhydrophobicity of the printed samples. a) Knife cutting, b) Quicksand impacting, c) Jetting, d) File abrasion, e) Sandpaper abrasion, f) Tape adhesion, and g) Fracture surface.

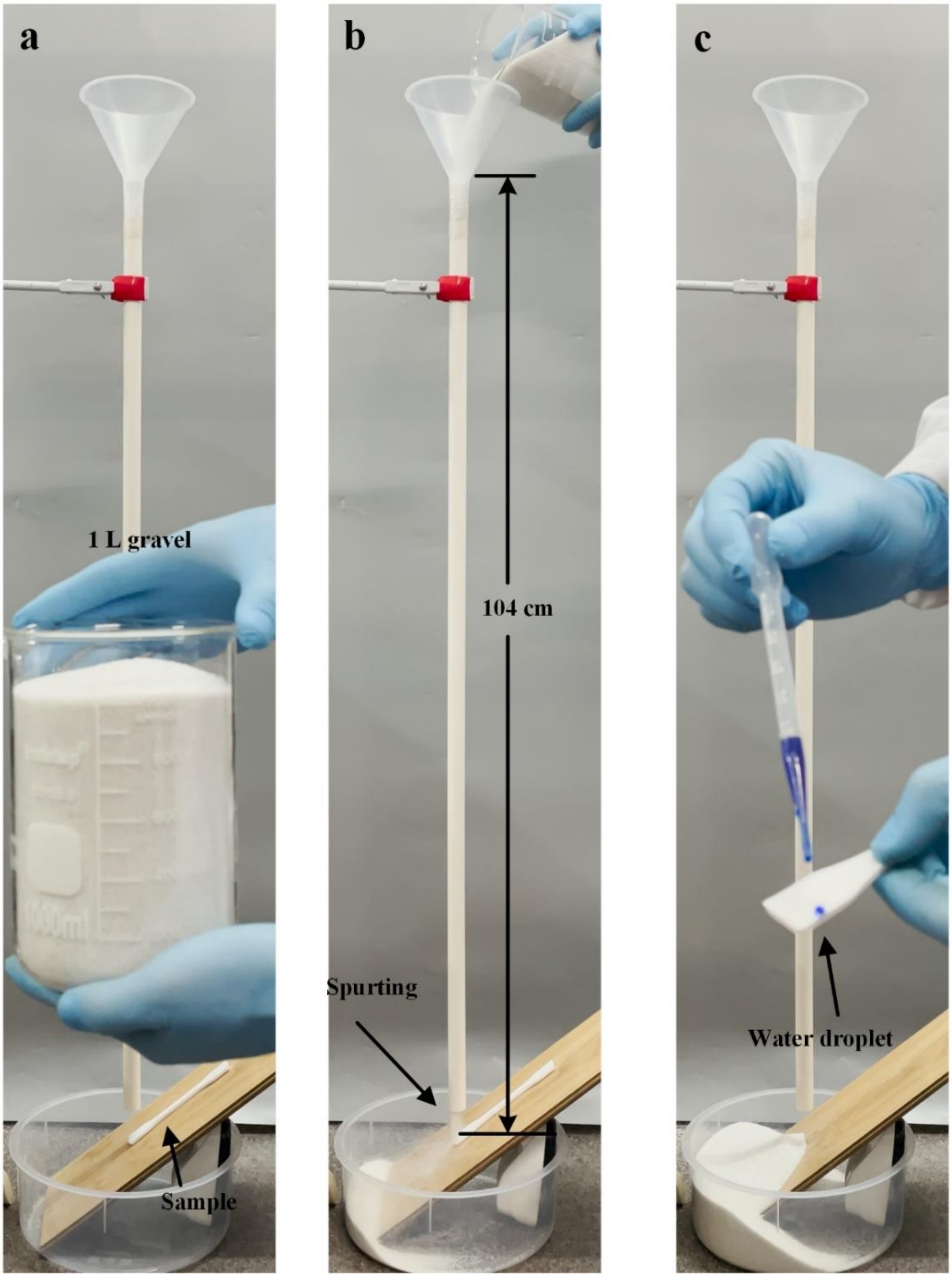

Figure S14. Quicksand impacting test. a-b) 1 L fine sand (diameters ≤0.6 mm) fell from a height of 104 centimeters through a vertical hollow tube, impacting onto the printed sample surface at an angle of 45°. The speed of the fine sand impacting the sample surface was ~4.5 m/s estimated by the gravimetric calculation. c) After quicksand impacting, several dyed water droplets were applied to the impacted surface to verify its superhydrophobicity. The printing parameters for this sample are 4 wt% HFS/PP weight ratio and a laser power density of 0.0725 J/mm^2.

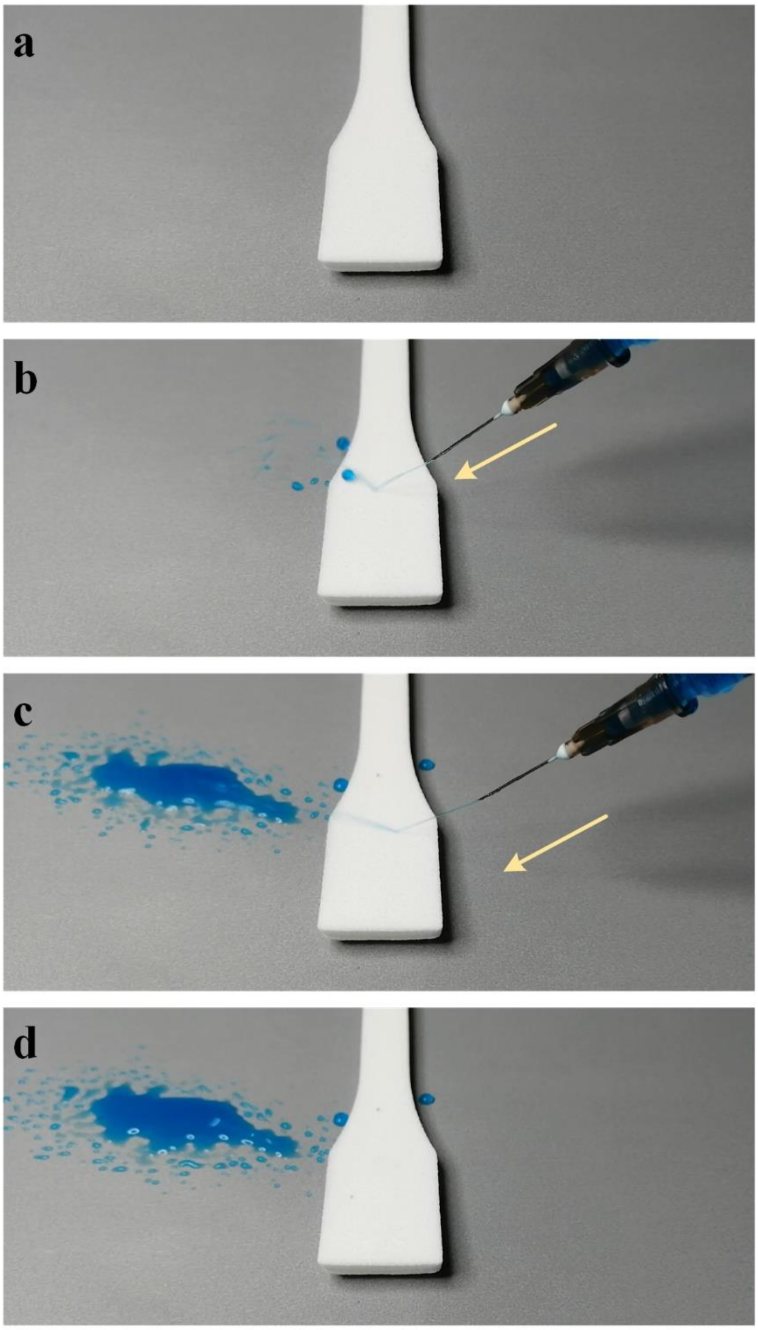

Figure S15. Jetting test. Photographs of the sample surfaces that remained clean after being impinged by a high-speed water flow extruded with a syringe. The printing parameters for this sample are 4 wt% HFS/PP weight ratio and a laser power density of 0.0725 J/mm^2.

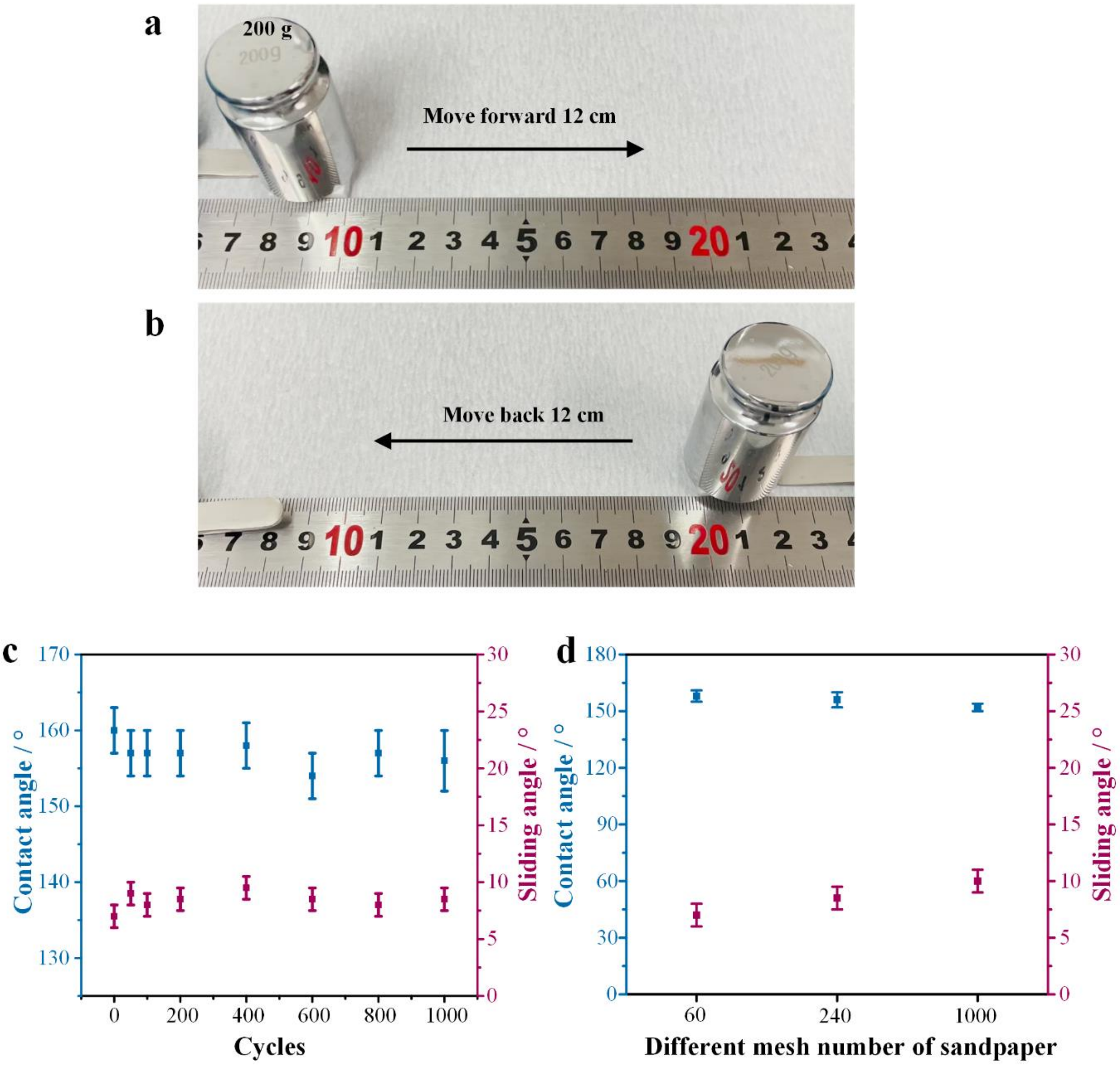

Figure S16. Sandpaper abrasion tests. a-b) Optical images of one-cycle sandpaper abrasion for the printed sample. c) Statistical diagram of water contact angles after the 50th, 100th, 200th, 400th, 600th, 800th and 1000th abrasion tests of sandpaper (Grit No. 240). d) Statistical diagram of water contact angles after 1000th abrasion tests of sandpapers with different grit numbers. The printing parameters for the samples are 4 wt% HFS/PP weight ratio and a laser power density of 0.0725 J/mm^2.

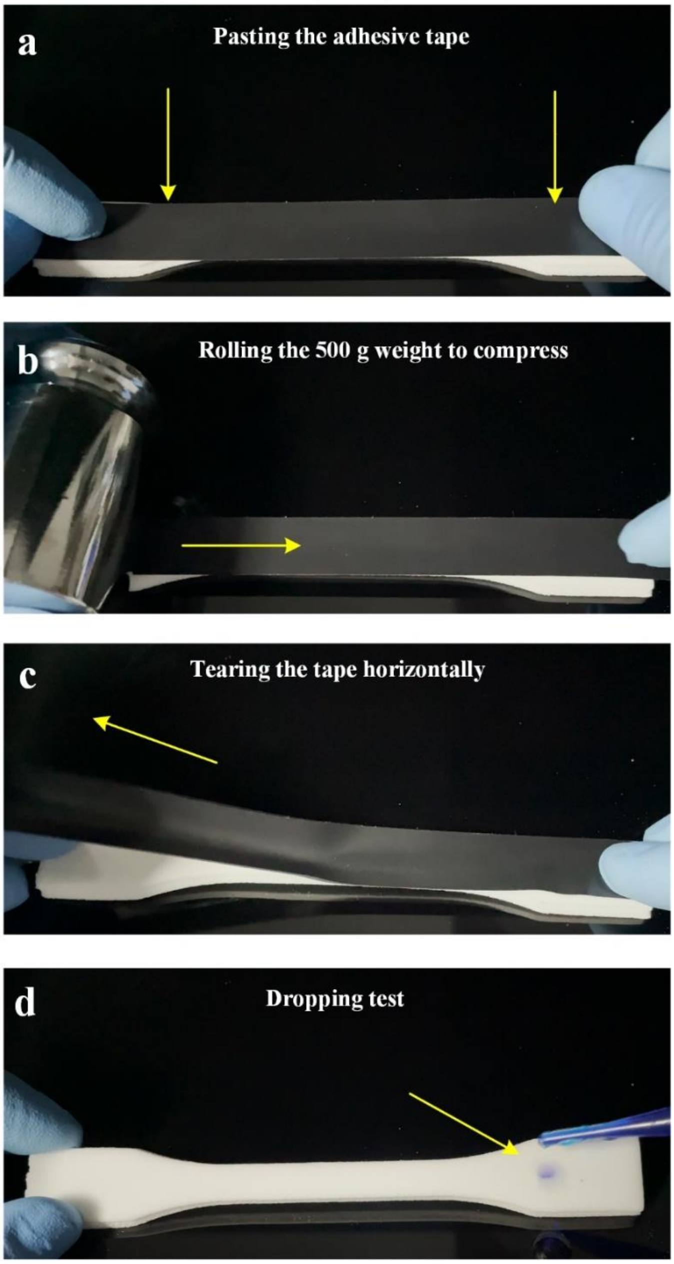

Figure S17. Tape test. a) First, place the printed flat sample horizontally on the table; b) Stick the tape on the surface of the sample and roll a weight of 500 g back and forth to make the tape fully bonded with the sample. c) Remove the tape by grabbing the free end of the tape and quickly pull it off (not jerked) at an angle of as close to 180° as possible; d) Finally, a few dyed water droplets were added to the taped sample to verify the superhydrophobic effect. The printing parameters for this sample are 4 wt% HFS/PP weight ratio and a laser power density of 0.0725 J/mm^2.

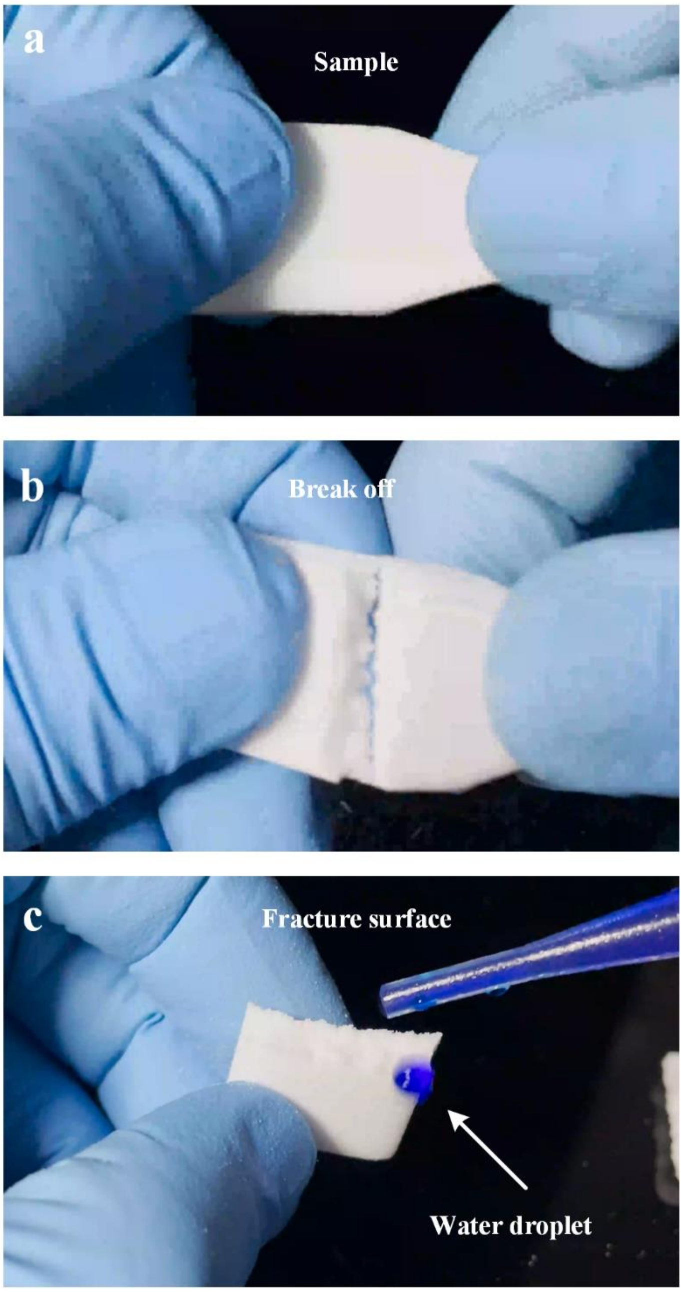

Figure S18. Fracture surface test. a-b) The fracture surface was obtained through totally breaking the printed sample by hands. c) A few dyed water droplets were added to the fracture surface to verify the superhydrophobic effect. The printing parameters for this sample are 4 wt% HFS/PP weight ratio and a laser power density of 0.0725 J/mm^2.

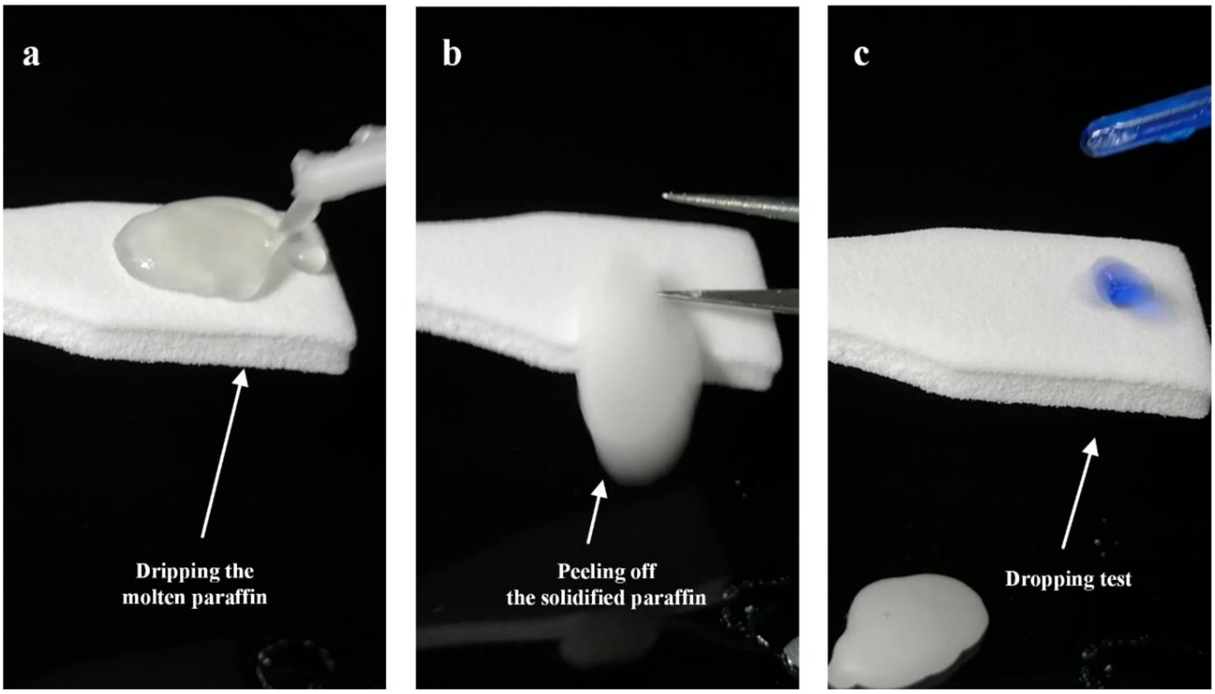

Figure S19. Oily residual test. a) Dropping the molten paraffin with ceresin on the printed sample surface. b) Removing the oily residual after they solidified. c) A few dyed water droplets were dropped to test the superhydrophobicity after removing the oily residual. The printing parameters for this sample are 4 wt% HFS/PP weight ratio and a laser power density of 0.0725 J/mm^2.

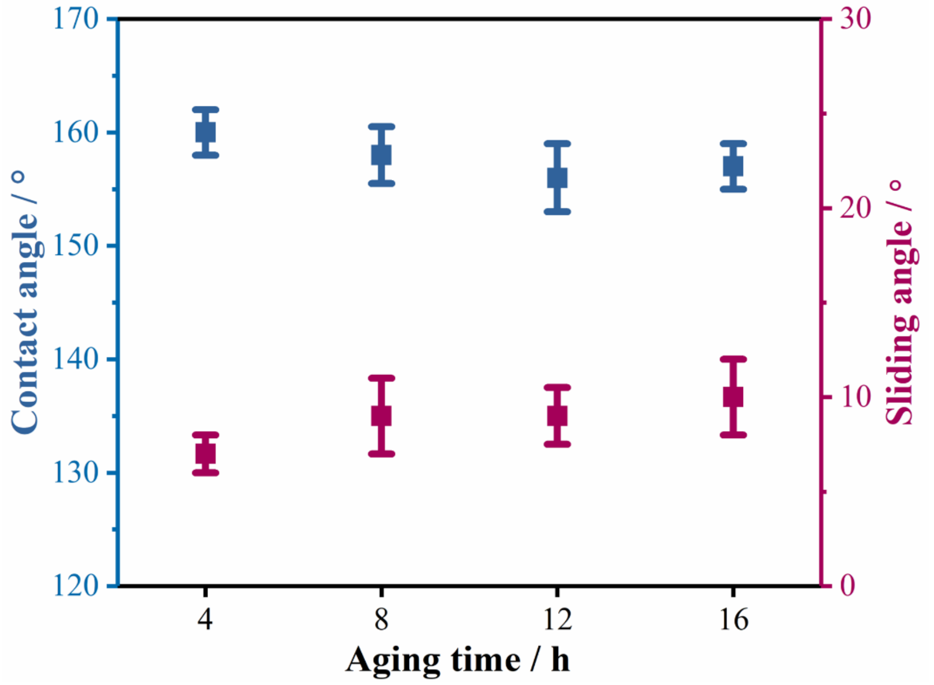

Figure S20. The relationship between the contact angle of the printed samples and their aging time. The printing parameters for this sample are 4 wt% HFS/PP weight ratio and a laser power density of 0.0725 J/mm^2.

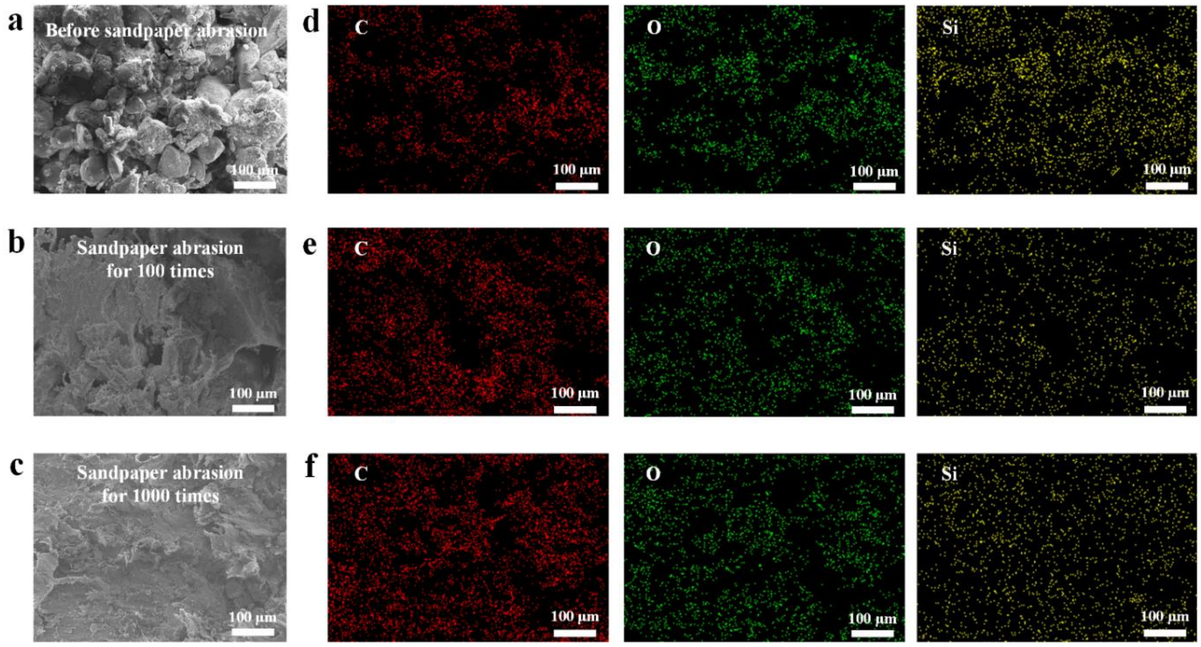

Figure S21. SEM images and their element analysis of the printed sample surfaces after diverse cycles of sandpaper test (Grit No. 240). a) SEM images and their element analysis of the surface before sandpaper abrasion; b-c) SEM images and their element analysis of the surfaces after 100th and 1000th sandpaper abrasion tests, respectively. The printing parameters for these samples are 4 wt% HFS/PP weight ratio and a laser power density of 0.0725 J/mm^2.

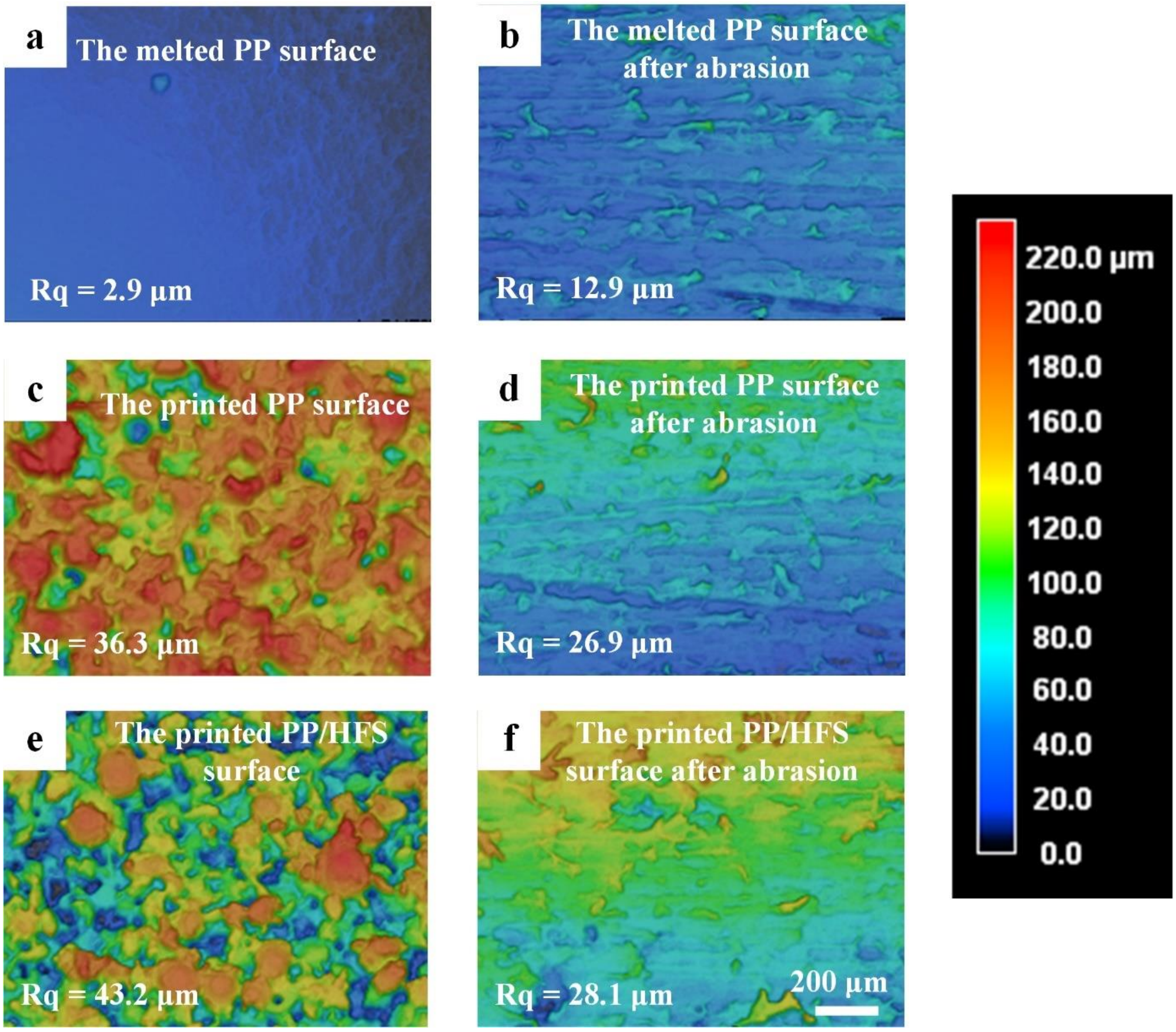

Figure S22. Comparison of 3D topographies of the melted PP surface, the printed PP surface and the printed PP/HFS surface before and after 1000th sandpaper abrasion.

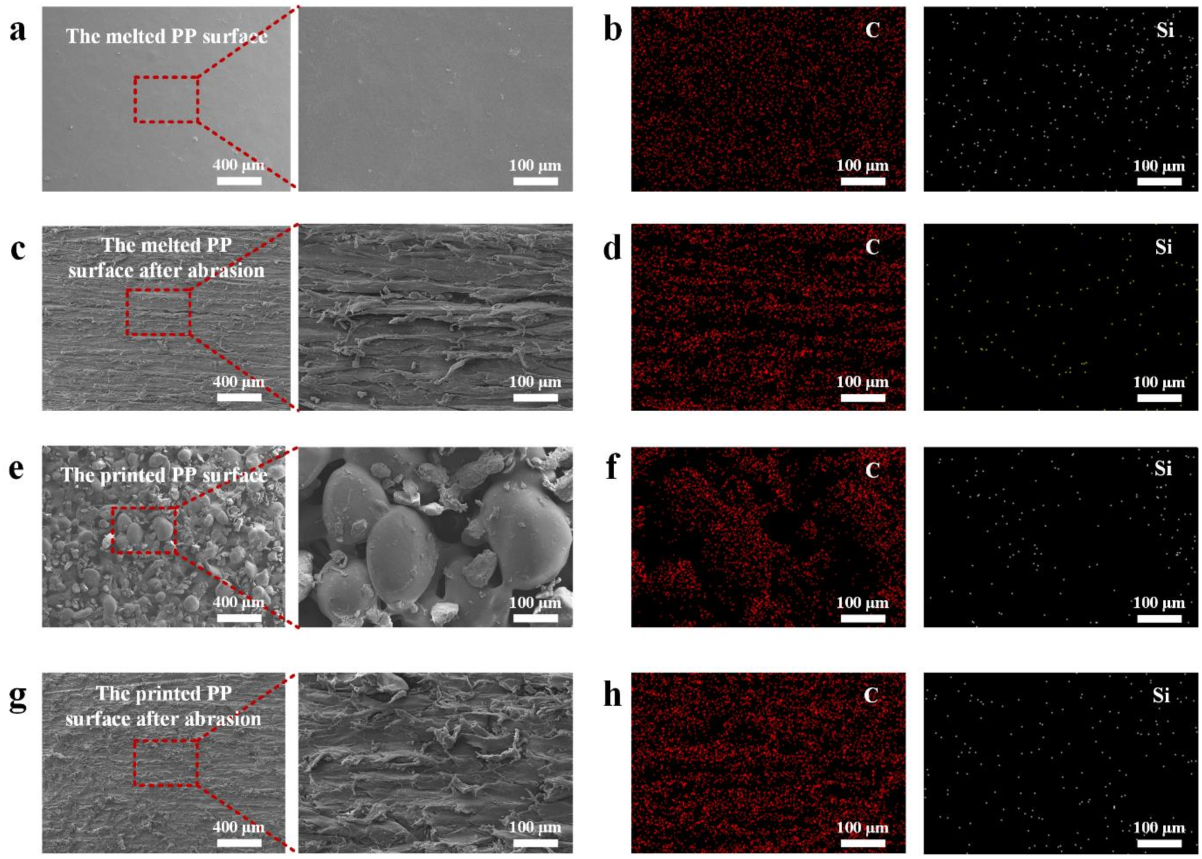

Figure S23. SEM images and element analysis of the melted PP surface and the printed PP surface before and after 1000th sandpaper abrasion.

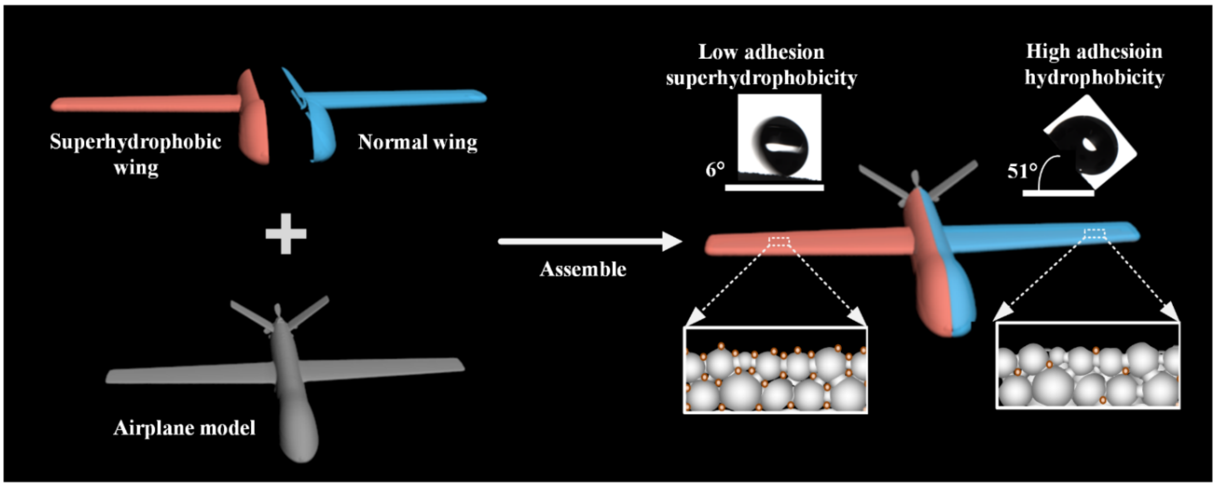

Figure S24. Schematic diagram of the airplane model assembled with the printed superhydrophobic and the normal wing shell. The superhydrophobic wing contains 4 wt% HFS and has a sliding angle of 6°. The normal wing is hydrophobic and has a sliding angle of 51°. The printing parameters for the superhydrophobic wing are 4 wt% HFS/PP weight ratio and a laser power density of 0.0725 J/mm^2. The printing parameters for the normal wing are 1 wt% HFS/PP weight ratio and a laser power density of 0.0350 J/mm^2.

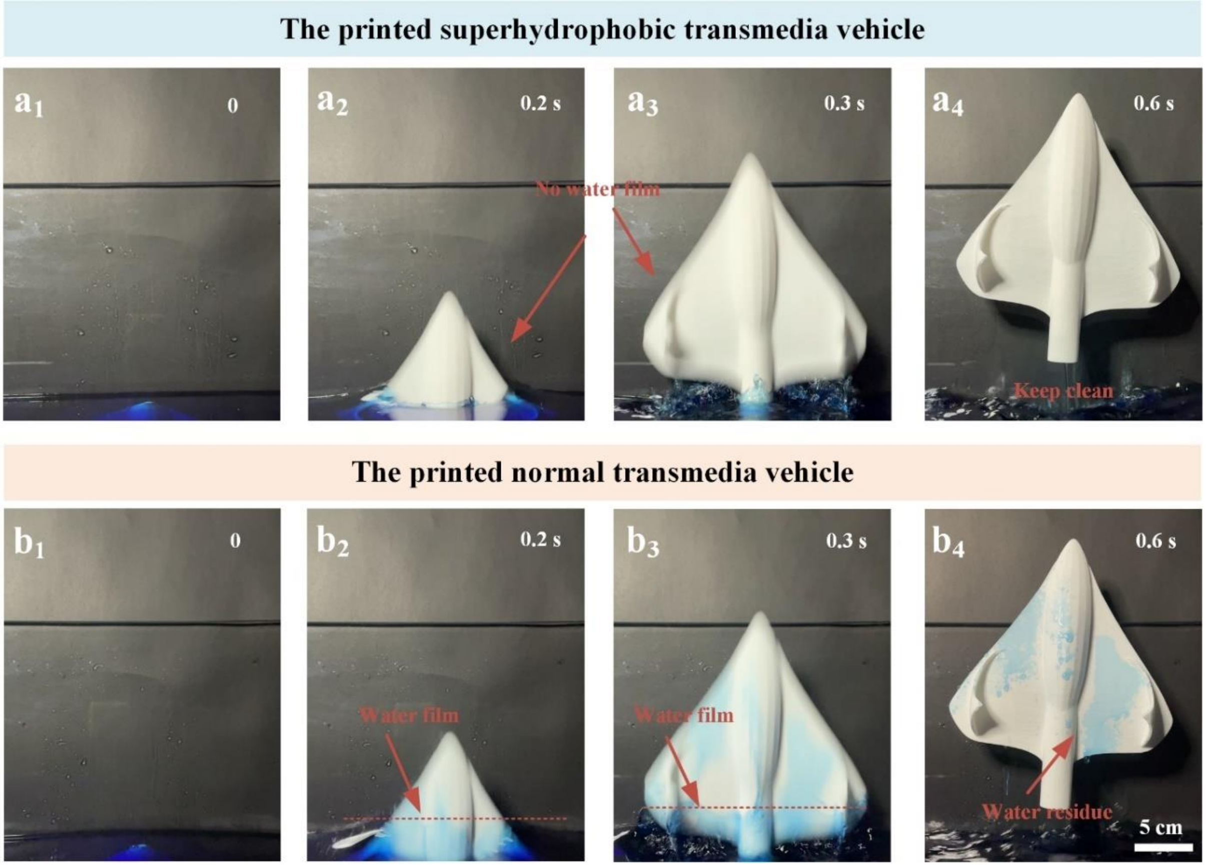

Figure S25. Time-lapse photographs of the printed superhydrophobic/normal transmedia vehicles flying out of the water. $a_{1\text{-}4}$) The printed superhydrophobic transmedia vehicle kept clean during the process of flying out of the water. The printing parameters for the superhydrophobic transmedia vehicle are 4 wt% HFS/PP weight ratio and a laser power density of 0.0725 J/mm^2. $b_{1\text{-}4}$) The printed normal transmedia vehicle had water film on the fuselage during flying out of the water, and kept water residue after leaving the water. The printing parameters for the normal transmedia vehicle are 1 wt% HFS/PP weight ratio and a laser power density of 0.0350 J/mm^2.

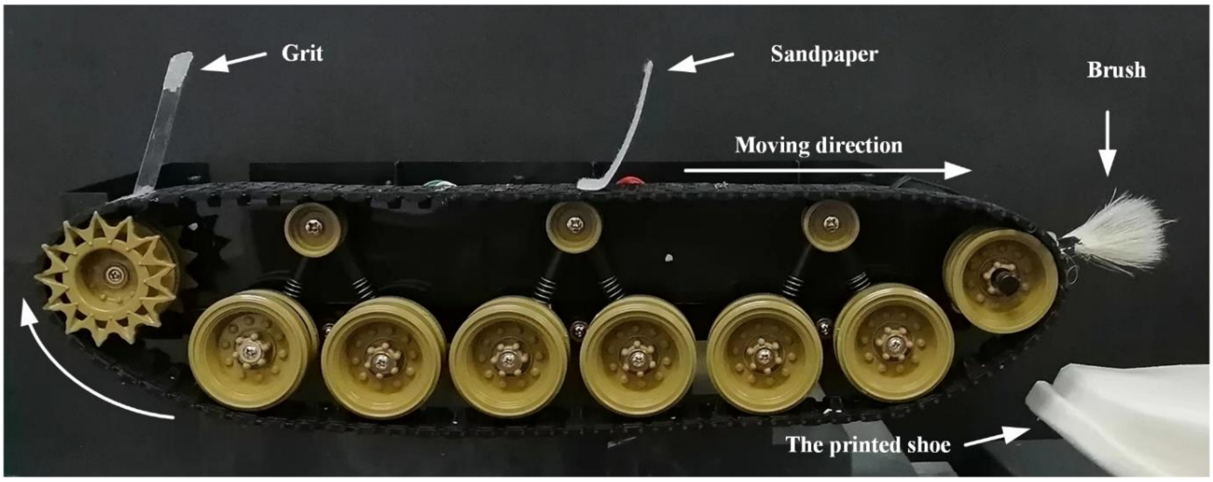

Figure S26. The home-made shoe polishing device. The home-made shoe polishing device includes of a motor, caterpillar band and three grinding units adhered on the caterpillar band. The three grinding units are brush, sandpaper and grit glued to PET film, respectively. When the motor starts, the three grinding units turn clockwise with the caterpillar band, polishing the printed shoe in sequence and continuously.

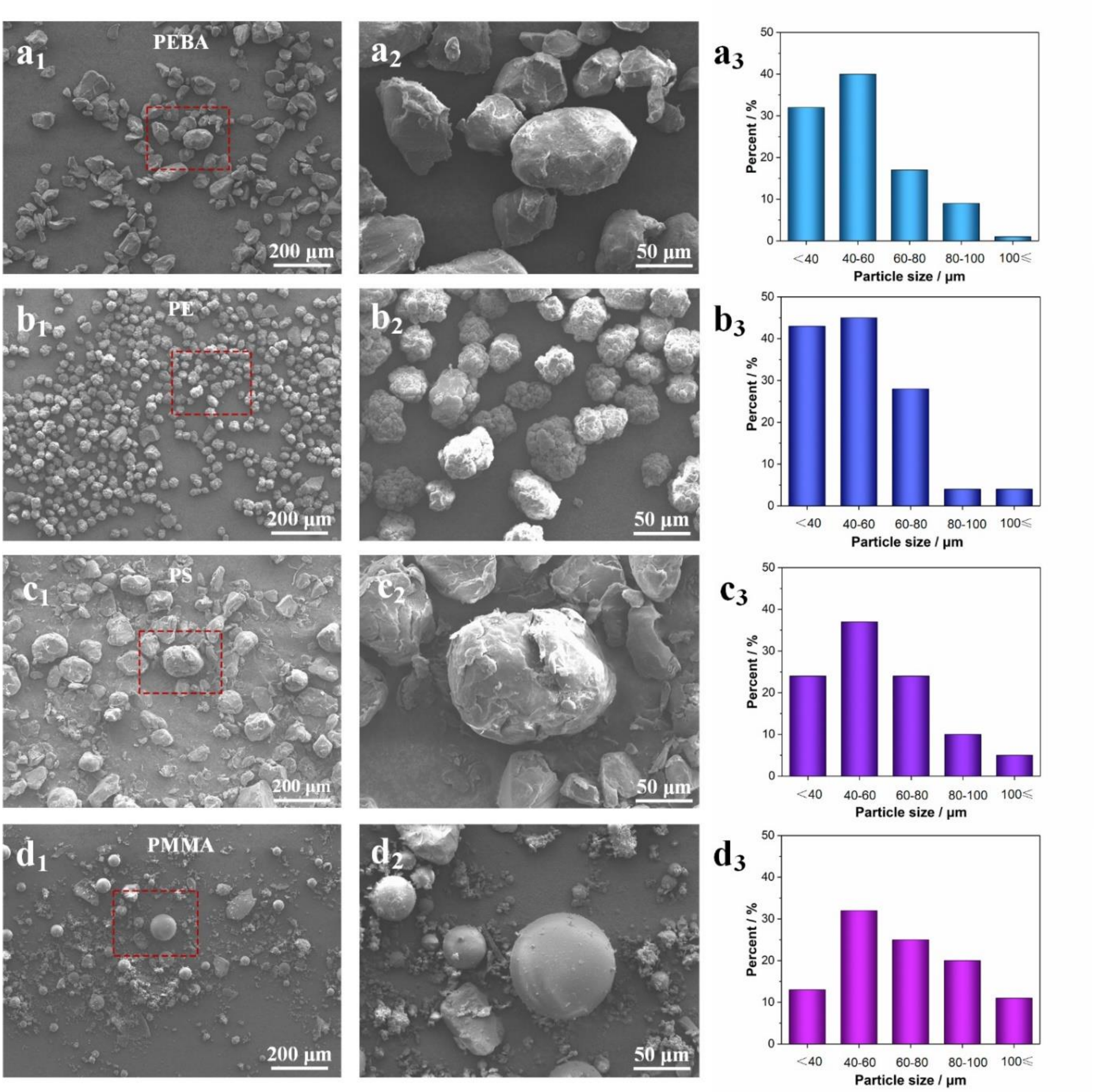

Figure S27. Characterization of the PEBA, PE, PS and PMMA grains and their size distribution. $a_{1\text{-}2}$) SEM images of PEBA grains. a_3) Statistics of PEBA particle size. $b_{1\text{-}2}$) SEM images of PE grains. b_3) Statistics of PE particle size. $c_{1\text{-}2}$) SEM images of PS grains. c_3) Statistics of PS particle size. $d_{1\text{-}2}$) SEM images of PMMA grains. d_3) Statistics of PMMA particle size.

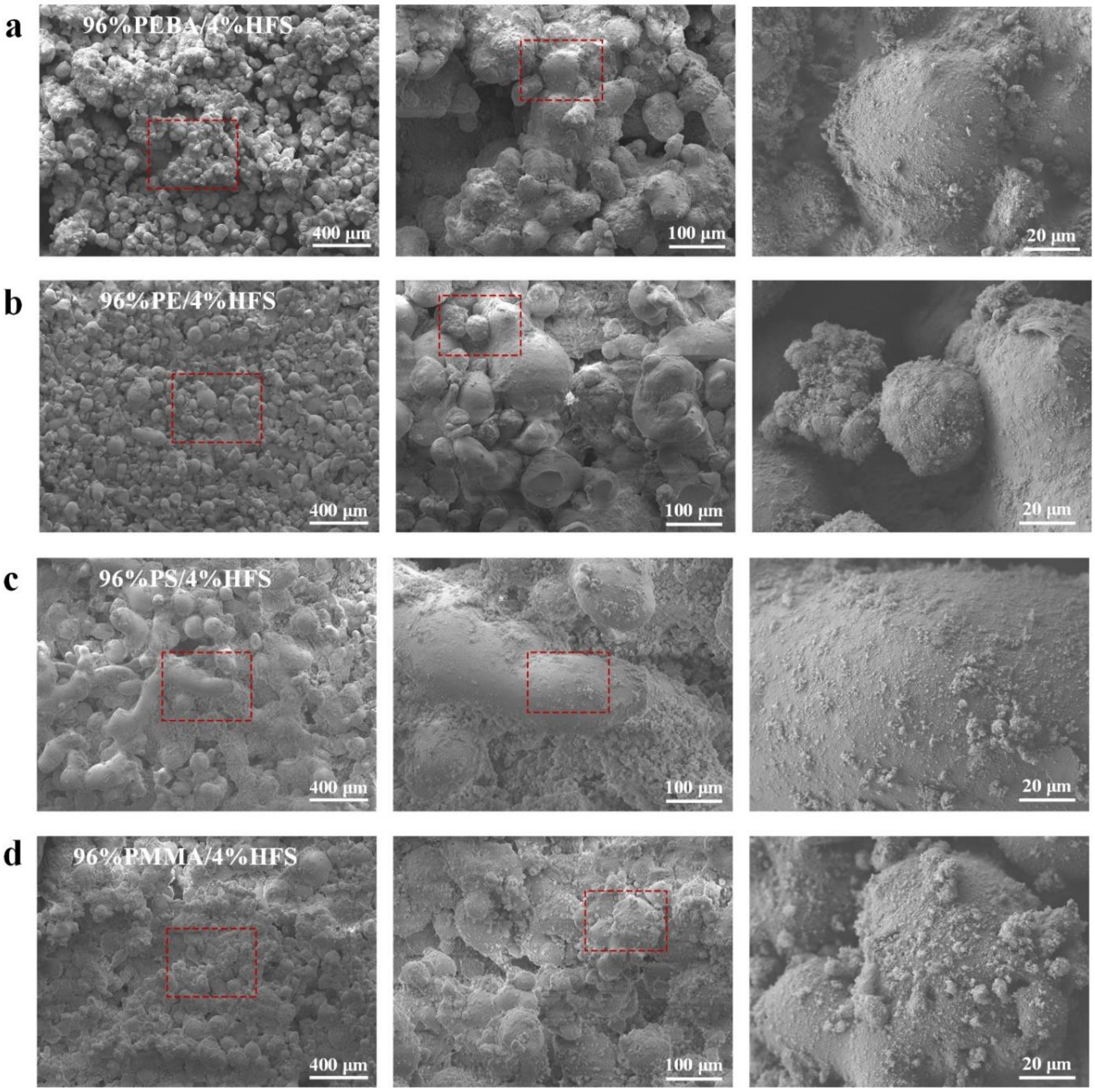

Figure S28. Top-view SEM images of the printed samples with different polymers. The HFS contents of all the samples are 4 wt%. The specific SLS processing parameters can be seen in Table S3.

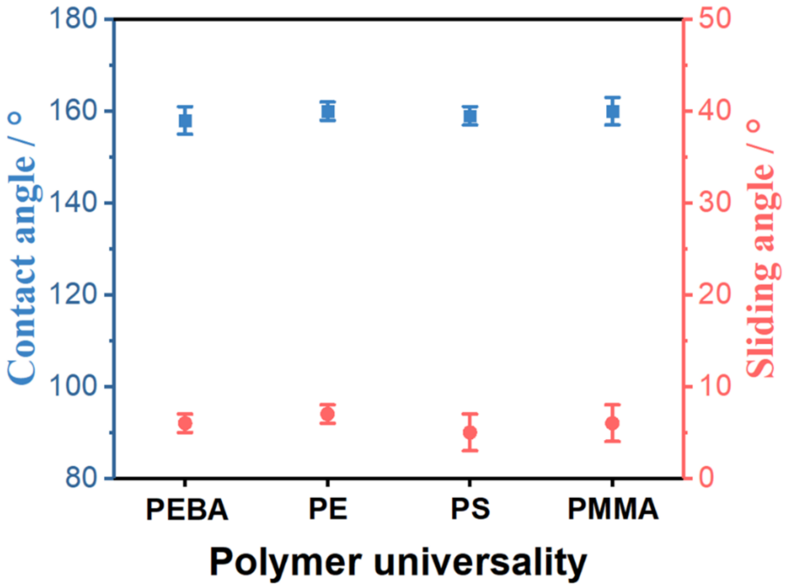

Figure S29. Polymer universality of this printing method of the abrasion-resistant superhydrophobic objects, including PEBA, PE, PS and PMMA. The HFS contents of all the samples are 4 wt%. The specific SLS processing parameters can be seen in Table S3.

Note S1. Details of the 3D printed superhydrophobic wing shell and transmedia vehicles.

The printed superhydrophobic wing shell and transmedia vehicle contain 4 wt% HFS and 96 wt% PP, and the processing parameters were the preheating temperature of 140 °C, the laser power of 29 W, the laser scan velocity of 4000 mm/s and the layer thickness of 0.1 mm. In order to better contrast the superhydrophobic effect, the normal wing shell and transmedia vehicle containing 1 wt% HFS and 99 wt% were printed. Their processing parameters were the preheating temperature of 140 °C, the laser power of 14 W, the laser scan velocity of 4000 mm/s and the layer thickness of 0.1 mm.

The printed superhydrophobic wing shell and the printed normal wing shell were fixed on the left and right wings of the airplane, respectively. Then, we used a sprayer to spray the aerosolized water onto the surface of the airplane. The results showed that the superhydrophobic wing on the left had almost no water residue left, while the normal wing on the right was covered with water droplets (Figure 4c and Movie S13).

The printed transmedia vehicles were magnetically controlled to fly out of the water at a speed of ~1 m/s. The superhydrophobic transmedia vehicle kept clean during the process of flying out of the water, while the normal transmedia vehicle had water film on the fuselage during flying out of the water, and kept water residue after leaving the water (Figure S23 and Movie S14).

Note S2. Details of the printed superhydrophobic shoe and the commercial shoe with superhydrophobic coating.

The printed superhydrophobic shoe contains 4 wt% HFS and 96 wt% PEBA, and the processing parameters were the preheating temperature of 120 °C, the laser power of 15 W, the laser scan velocity of 2000 mm/s and the layer thickness of 0.12 mm. The commercial shoe with superhydrophobic coating was prepared by spraying superhydrophobic coatings on the commercial shoe. The superhydrophobic coating solution was obtained by ultrasonic dispersion of 1 g of HFS in 100 mL ethanol.

In order to illustrate the mechanical stability of the printed superhydrophobic shoe, a home-made shoe polishing device including of a motor, caterpillar band and three grinding units adhered on the caterpillar band was prepared (Figure S24). The superhydrophobic effects of the printed superhydrophobic shoe remained even after 1000th wear with sandpaper, brush and grit (Figure 4d). However, the commercial shoe with superhydrophobic coating lost its superhydrophobic effect after dozens of sanding (Figure 4e and Movie S15).

Table S1. The dependence of wettability of the printed objects on the laser power density and the HFS content.

PP/HFS \ *Power/J/mm^2*	**0.0350**	**0.0425**	**0.0500**	***0.0575***	**0.0650**	**0.0725**
**100/0**	☆	×	×	×	×	×
**99/1**	☆	☆	×	×	×	×
**98/2**	△	△	△	×	×	×
**97/3**	×	×	△	△	△	△
**96/4**	×	×	√	√	√	√
**95/5**	×	×	√	√	√	√

☆：It can be printed but not superhydrophobic.

△：It can be printed. The samples are superhydrophobic with high adhesion.

√： It can be printed. The samples are superhydrophobic with low adhesion.

×: Continuous and smooth printing cannot be carried out, and the problem of edge warping or low mechanical strength after printing often occurred.

Table S2. Comparison of sliding angles and sizes of the printed superhydrophobic objects in this study and existing reports.

**Ref.**	**Printing method**	**Sliding angle**	**Printing size**
**Ref. 10**	Immersed surface accumulation 3D (ISA-3D) printing	180°	0.4 cm
**Ref. 11**	Projection microstereolithography (PμSL) based 3D printing	180°	1.5 cm
**Ref. 12**	Digital light processing (DLP) 3D printing	5°	2.0 cm
**Ref. 13**	3D micro-printing (μ-printing) method based on digital ultraviolet lithography	＜10°	0.2 cm
**Ref. 14**	Two-photon polymerization (TPP)-based 3D printing	180°	3.0 cm
**Ref. 23**	Two-photon polymerization (TPP)-based 3D printing	180°	2.0 cm
**Ref. 26**	Initiator integrated 3D printing (i3DP)	＜10°	2.5 cm
**Ref. 27**	Laser lithography	180°	0.8 cm
**Ref. 28**	Digital Light Processing (DLP) 3D printing	3°	1.5 cm
**This study**	**Selective laser sintering (SLS) 3D printing**	**6°**	**50.0 cm**

Table S3. The SLS processing parameters for polymer universality, including PEBA, PE, PS and PMMA. The mass fraction of all polymers was 96 wt% and the mass fraction of HFS was 4 wt%.

**Parameter**	**Preheating temperature / °C**	**laser power / W**	**laser scan velocity / mm/s**	**layer thickness / mm**
**PEBA/HFS**	120	15	2000	0.12
**PE/HFS**	120	30	2500	0.10
**PS/HFS**	78	30	2500	0.12
**PMMA/HFS**	100	25	2200	0.12

References

[10] Y. Yang, X. Li, X. Zheng, Z. Chen, Q. Zhou and Y. Chen, *Adv. Mater*. **2018**, *30*, 1704912.

[11] Q. Yin, Q. Guo, Z. Wang, Y. Chen, H. Duan and P. Cheng, *ACS Appl. Mater. Interfaces,* **2021**, *13*, 1979-1987.

[12] Z. Dong, M. Vuckovac, W. Cui, Q. Zhou, R. H. A. Ras and P. A. Levkin, *Adv. Mater*. **2021**, *33*, e2106068.

[13] Y. Zhang, M.-J. Yin, X. Ouyang, A. P. Zhang and H.-Y. Tam, *Appl. Mater. Today* **2020**, *19*, 100580.

[14] B. Wang, J. Chen, C. Kowall and L. Li, *ACS Appl. Mater. Interfaces* **2020**, *12,* 35725-35730.

[23] X. Liu, H. Gu, H. Ding, X. Du, Z. He, L. Sun, J. Liao, P. Xiao and Z. Gu, *Small* **2019**, *15,* e1902360.

[26] X. Wang, X. Cai, Q. Guo, T. Zhang, B. Kobe and J. Yang, *Chem. Commun.* **2013**, *49,* 10064-10066.

[27] M. I. Abid, L. Wang, Q.-D. Chen, X.-W. Wang, S. Juodkazis and H.-B. Sun, *Laser Photonics Rev.* **2017**, *11,* 1600187.

[28] G. Kaur, A. Marmur and S. Magdassi, *Addit. Manuf.* **2020**, *36,* 101669.
